# Engineering of versatile redox partner fusions that support monooxygenase activity of functionally diverse cytochrome P450s

**DOI:** 10.1038/s41598-017-10075-w

**Published:** 2017-08-29

**Authors:** Patrick J. Bakkes, Jan L. Riehm, Tanja Sagadin, Ansgar Rühlmann, Peter Schubert, Stefan Biemann, Marco Girhard, Michael C. Hutter, Rita Bernhardt, Vlada B. Urlacher

**Affiliations:** 1 Institute of Biochemistry, Heinrich-Heine University Düsseldorf, Universitätsstr. 1, 40225 Düsseldorf, Germany; 2 Center for Bioinformatics, Saarland University, Campus Building E2.1, 66123 Saarbrücken, Germany; 3 Institute of Biochemistry, Saarland University, Campus Building B2.2, 66123 Saarbrücken, Germany

## Abstract

Most bacterial cytochrome P450 monooxygenases (P450s or CYPs) require two redox partner proteins for activity. To reduce complexity of the redox chain, the *Bacillus subtilis* flavodoxin YkuN (Y) was fused to the *Escherichia coli* flavodoxin reductase Fpr (R), and activity was tuned by placing flexible (GGGGS)_n_ or rigid ([E/L]PPPP)_n_ linkers (n = 1–5) in between. P-linker constructs typically outperformed their G-linker counterparts, with superior performance of YR-P5, which carries linker ([E/L]PPPP)_5_. Molecular dynamics simulations demonstrated that ([E/L]PPPP)_n_ linkers are intrinsically rigid, whereas (GGGGS)_n_ linkers are highly flexible and biochemical experiments suggest a higher degree of separation between the fusion partners in case of long rigid P-linkers. The catalytic properties of the individual redox partners were best preserved in the YR-P5 construct. In comparison to the separate redox partners, YR-P5 exhibited attenuated rates of NADPH oxidation and heme iron (III) reduction, while coupling efficiency was improved (28% *vs*. 49% coupling with *B. subtilis* CYP109B1, and 44% *vs*. 50% with *Thermobifida fusca* CYP154E1). In addition, YR-P5 supported monooxygenase activity of the CYP106A2 from *Bacillus megaterium* and bovine CYP21A2. The versatile YR-P5 may serve as a non-physiological electron transfer system for exploitation of the catalytic potential of other P450s.

## Introduction

Cytochrome P450 monooxygenases (CYPs or P450s) are highly versatile heme containing enzymes that catalyse a wide variety of oxidation reactions, while accepting a large diversity of substrates. The impressive range of reactions catalysed by P450s includes amongst others hydroxylation, epoxidation, dealkylation and deamination, as well as unusual reactions such as aromatic dehalogenation and Baeyer-Villiger oxidation^1–3^. Illustrative of their diverse biological functions, P450s are capable of converting amongst others fatty acids, steroids, prostaglandins, terpenes, and xenobiotics such as drugs and antibiotics^4, 5^. It is therefore not surprising that these multipurpose biocatalysts have become attractive targets for application in biotechnology and synthetic biology^6^.

P450s catalyse the reductive scission of molecular oxygen upon which one oxygen atom is introduced into the non-activated substrate, while the second atom is reduced to water^7^. Activation of molecular oxygen relies on the successive delivery of two electrons derived from the pyridine cofactor NAD(P)H to the heme iron, which is typically facilitated by a dedicated redox partner system^8, 9^.

Based on the number of proteins involved, two major types of P450 redox systems can be distinguished: (i) two-component systems consisting of an FAD-containing reductase and either an iron-sulfur containing ferredoxin or an FMN-containing flavodoxin. These systems are commonly found in bacteria and mitochondria, and (ii) a single diflavin (FAD/FMN) P450 reductase (CPR) that supports the function of eukaryotic microsomal P450s. A limited number of bacterial P450s have been identified that carry such a CPR-like module as a fused functional domain (for example CYP102A1 from *Bacillus subtilis*)^8, 9^.

The intrinsic dependence on NAD(P)H and redox partner(s) however limits the application and development of P450s as biocatalysts^10^. Moreover, identification of the physiological redox partner(s) of a given P450 is often hampered by the fact that host genomes usually contain numerous candidate genes coding for electron transfer proteins, which mostly are not located near the P450 gene(s)^8, 10^. Therefore surrogate electron transfer systems are frequently used for functional characterization and/or biocatalytic application of P450s. Cross-reactivity of eukaryotic P450s has been successfully exploited to support catalysis of a variety of mammalian enzymes^11^, whereas two-component systems are frequently used to support the function of P450s from microorganisms. Notorious examples of these latter systems include putidaredoxin reductase (PdR) and putidaredoxin (Pdx) from *Pseudomonas putida* ^12, 13^, bovine adrenodoxin reductase (AdR) and adrenodoxin (Adx)^14, 15^ and flavodoxin/ferredoxin reductase (Fpr) from *E. coli* combined with either the flavodoxin FldA from *E. coli* or the flavodoxin YkuN from *Bacillus subtilis* ^16, 17^. Moreover, in several cases electron transfer proteins could be covalently joined or even functionally attached to a P450^18–25^. The principal aims of creating such artificial fusion constructs are to reduce complexity of the P450 systems and to improve the electron transfer properties to enhance catalytic performance^11, 26^.

Here, we report the construction of a set of redox fusion enzymes in which the flavodoxin YkuN from *B. subtilis* is fused to the N-terminus of the flavodoxin reductase Fpr from *E. coli*. To tune activity of the fused redox partners the linker region between them was varied in a systematic manner. Herein, both flexible (GGGGS)_n_ and rigid ([E/L]PPPP)_n_ linkers of different lengths (n = 1–5) were introduced between YkuN and Fpr by using the previously established DuaLinX tool^19^. After expression in *E. coli* and subsequent purification, the activity of the fusion constructs was investigated with respect to their ability to oxidize NADPH and reduce the P450 heme iron, as well as their capability to support the monooxygenase activity of a variety of P450s *in vitro*.

## Results

### Construction of redox partner fusion enzymes consisting of *B. subtilis* YkuN and *E. coli* Fpr

**Figure 1:**
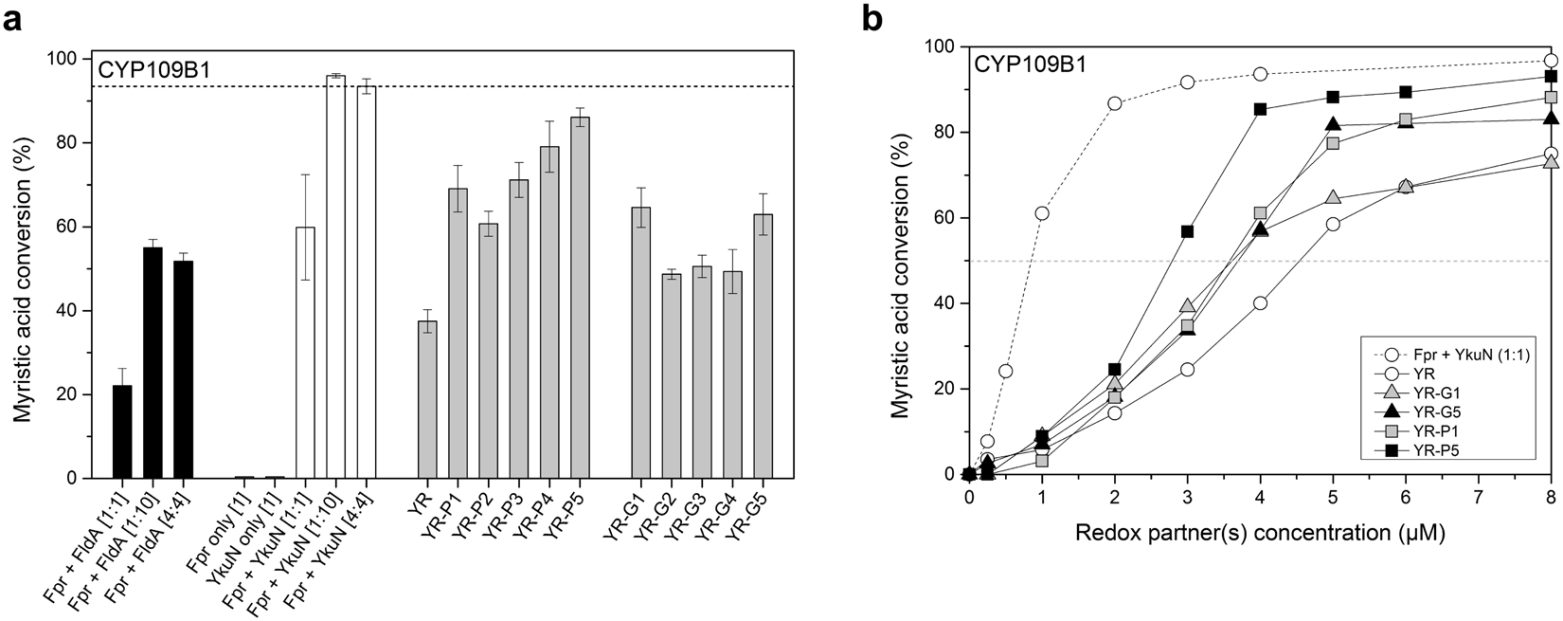
Myristic acid conversion by *B. subtilis* CYP109B1 supported by different redox partners. Reactions were started by the addition of a mixture of NADPH and myristic acid and allowed to proceed for 120 min under the support of an NADPH regenerating system. (**a**) CYP109B1 (1 µM) conversion reactions were carried out in the presence of non-fused redox partners Fpr and FldA (black bars), Fpr and YkuN (white bars) or with different YkuN-Fpr fusion constructs (grey bars). Reductase (Fpr) and flavodoxin (FldA or YkuN) together with CYP109B1 were employed at respective ratios of 1:1:1, 1:10:1 and 4:4:1. Reactions conducted with YkuN-Fpr (YR) fusion constructs and CYP109B1 were carried out at a respective ratio of 4:1. YR indicates the linker-less YkuN-Fpr fusion construct, whereas linker designations P1 - P5 and G1 - G5 correspond to linker sequences (GGGGS)_n_ and ([E/L]PPPP)_n_ of different lengths (n = 1–5). The data presented are average values of 3–6 independent conversion reactions with indicated standard deviation. (**b**) Myristic acid conversion by CYP109B1 in the presence of different concentrations of selected fusion constructs or non-fused Fpr/YkuN. The ratio of non-fused redox partners was maintained at 1:1.

The flavodoxin YkuN from *Bacillus subtilis* is a promiscuous electron carrier capable of transferring electrons to a variety of P450s, including endogenous CYP107H1 (P450 BioI) and CYP109B1, but also to heterologous P450s such as CYP154A8 from *Nocardia farcinica* or CYP154E1 from *Thermobifida fusca* YX^16, 17, 27^. Herein, YkuN is typically paired with the flavodoxin reductase Fpr from *E. coli*, which provides the NADPH-derived electrons. The “mixed” redox pair Fpr/YkuN often outperforms physiological redox pairs such as *E. coli* Fpr/FldA or *P. putida* PdR/Pdx^16, 17^. Consistent with this, Fpr/YkuN supported substantially higher conversion of myristic acid by CYP109B1 than the Fpr/FldA system (Fig. 1a).

Importantly, even in the case of natural P450 redox chains, such as PdR:Pdx:P450cam, excess of the ferredoxin Pdx is required for productive catalysis *in vitro* ^28^. Similarly, for Fpr/YkuN/P450 systems, a respective stoichiometry of 1:10:1 is usually sufficient to efficiently drive P450-mediated catalysis *in vitro* ^16, 17^. Thus surplus of flavodoxin or ferredoxin overcomes apparent rate-limiting steps in electron transfer^16, 28, 29^. In support of this, a 1:10:1 Fpr/YkuN/CYP109B1 system achieved 36% higher conversion of myristic acid than a 1:1:1 system (Fig. 1a), which is consistent with the function of YkuN as an electron shuttle that transfers the NADPH-derived electrons from the reductase to the P450^27, 29^. A stimulatory effect was also noted for the alternative system using the *E. coli* flavodoxin FldA (Fig. 1a). Nevertheless, independent of the stoichiometry applied the Fpr/YkuN redox pair proved to be superior to Fpr/FldA in supporting CYP109B1 catalysis, which makes YkuN a promising candidate for covalent fusion to Fpr.

YkuN was fused to the N-terminus of Fpr, leaving the C-terminus of Fpr free, as it was demonstrated previously that attachment of FldA to the C-terminus of Fpr leads to reduced enzyme function, whereas constructs with the fusion partners in reversed order were substantially more active^19^. Attachment of the flavodoxin to the C-terminus of Fpr likely interferes with the function of the aromatic residues at the extreme C-terminus of Fpr, which are important for NADPH binding and electron exchange^30–32^. It is worthy of note that homologous catalytic residues within the reductase domain of natural fusion proteins such as CPR and self-sufficient P450s such as CYP102A1 are also found at the C-terminus^8, 9^.

To optimize activity of the YkuN-Fpr (YR) fusion construct both flexible (GGGGS)_n_ and rigid ([E/L]PPPP)_n_ linkers of different lengths (n = 1–5) were placed between the fusion partners (for linker specifications see Supplementary Table S2). Thus, 11 different fusion constructs were created that were expressed in *E. coli*. The corresponding fusion proteins were subsequently isolated from the soluble protein fraction and purified to near homogeneity by IMAC (Supplementary Fig. S1), typically yielding 1–2 mg of purified fusion protein from 40 ml culture (OD_600_ = 4–5).

### Ykun-Fpr fusion constructs support monooxygenase activity of CYP109B1

The ability of the fusion constructs (4 µM) to support P450 monooxygenase activity was tested *in vitro* with CYP109B1 (1 µM) using myristic acid as substrate (Fig. 1a). Because the stoichiometry of the fused redox partners is fixed at 1:1, control reactions were conducted with 4 µM each of non-fused Fpr and YkuN. Independent of the flavodoxin used (FldA or YkuN), myristic acid conversion achieved with the 4:4:1 reconstituted system was nearly equal to that obtained with the commonly used 1:10:1 system (Fig. 1a). Importantly, the different fusion constructs were all able to support CYP109B1 catalysis (Fig. 1a), which indicates that the covalently attached Fpr and YkuN have retained their ability to deliver the necessary electrons to CYP109B1. The linker-less fusion YR exhibited appreciable activity, supporting ~38% conversion of myristic acid (Fig. 1). Nevertheless, conversion was substantially lower than with equivalent amounts of non-fused Fpr/YkuN, suggesting reduced functionality upon direct attachment of the redox partners. Introduction of a linker between YkuN and Fpr however substantially improved activity. In particular the fusion constructs YR-P4 and YR-P5 supported high myristic acid conversion (79% and 86%, respectively), which is close to the 94% conversion achieved with non-fused Fpr/YkuN (Fig. 1a). Quantitative product analysis revealed that both the regioselectivity of CYP109B1 for myristic acid hydroxylation as well as product distribution was not affected by the fusion constructs (Supplementary Table S3). In all cases, the carbon atoms C-11 (ω_−3_) and C-12 (ω_−2_) of myristic acid were preferentially hydroxylated.

Interestingly, the fusion constructs carrying proline-rich linkers (P1 - P5) in all cases outperformed their glycine-rich (G1–G5) counterparts (Fig. 1a). Moreover, the performance of the P-linker constructs seems to be dependent on the linker length, supporting higher conversion with increased linker length, whereas for the G-linker constructs a dependence on linker length was not evident (Fig. 1a).

Spectral analysis of the different YkuN-Fpr fusion constructs revealed nearly identical absorbance characteristics (Supplementary Fig. S3), which indicates that the fusion proteins have a similar cofactor content (Fpr-FAD and YkuN-FMN) as well as a similar cofactor-protein environment. It seems therefore unlikely that the differences in activity between the fusion constructs relate to linker induced effects on cofactor binding or protein folding.

The performance of the fusion constructs was further investigated using different redox protein concentrations (0–8 µM). In all cases, sigmoid curves were obtained over the range of concentrations tested (Fig. 1b). The dependence of CYP109B1 activity on the fusion protein concentration was clearly different from that of an equivalent system of non-fused Fpr/YkuN maintained at a 1:1 ratio, and also differed among the fusion constructs, which indicates rather complex behaviour (Fig. 1b).

Overall, YR-P5 proved the most effective fusion construct, as it supported nearly complete conversion of myristic acid at protein concentrations of 4–8 µM, much like the non-fused redox partners. However, at concentrations <4 µM, the performance of YR-P5 dropped well below that of the non-fused Fpr/YkuN, as was also observed for the other tested fusion constructs (Fig. 1b). As a measure for the overall performance of the redox partners, the concentration of the redox enzymes at which 50% of myristic acid (EC_50_) was converted by CYP109B1 was estimated from Fig. 1b. The approximate EC_50_ are 0.8 µM for non-fused Fpr/YkuN, 2.7 µM for YR-P5, 3.5 µM for both YR-P1 and YR-G1, 3.6 µM for YR-G5 and 4.5 µM for the linker-less construct YR, which demonstrates that YR-P5 is the superior fusion construct.

### Intrinsic properties of the (GGGGS)_n_ and ([E/L]PPPP)_n_ linkers

**Figure 2:**
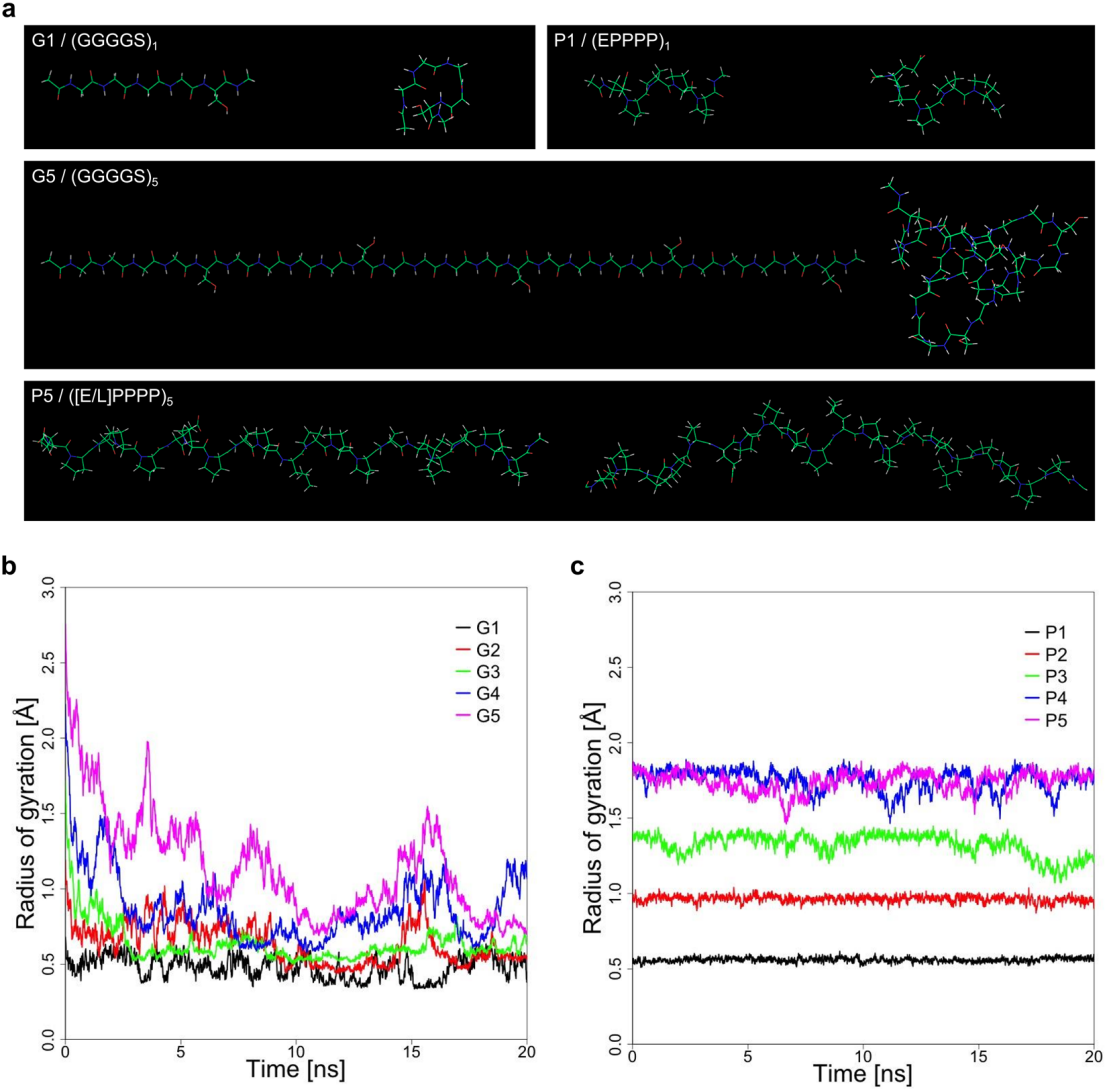
MD simulations of the (GGGGS)_n_ and ([E/L]PPPP)_n_ linkers used to functionally connect YkuN and Fpr. (**a**) Structural snapshots of (GGGGS)_n_ and ([E/L]PPPP)_n_ linkers of length 1 and 5. Depicted in each panel are the starting conformations (*left*) and the lowest energy conformation found during the 20 ns MD simulations (*right*). Development of the radius of gyration (Rg) during MD simulations for (GGGGS)_n_ and ([E/L]PPPP)_n_ linkers of lengths (n = 1–5) are shown in (**b**) and (**c**), respectively. The strong decrease in Rg for (GGGGS)_n_ linkers reflects the hydrophobic collapse, whereas the ([E/L]PPPP)_n_ linkers remain in an extended conformation.

To further evaluate the properties of the (GGGGS)_n_ and ([E/L]PPPP)_n_ linkers molecular dynamics (MD) simulations were carried out. MD simulations of the G-linkers revealed that starting from a linear extended conformation these linkers readily fold into more compact random structures (Fig. 2a). Collapse of the extended conformation is particularly evident for linkers G3, G4 and G5, which consist of 3, 4 and 5 (GGGGS)-repeats, respectively. These linkers exhibited a substantial (>1 Å) decrease in the radius of gyration (Rg) over time (Fig. 2b). Moreover, the Rg of the G4 and G5 linker exhibited large oscillations over time, which indicates substantial structural rearrangements and high structural flexibility. Structural behaviour of the ([E/L]PPPP)_n_ linkers on the other hand was clearly different. The Rg of the different P-linkers remained largely constant over time, with only minor oscillations observed for linkers P3–P5 (Fig. 2c). Thus, the P-linkers effectively maintained their linear conformation. Consequently, the Rg of the P-linkers exhibited a dependence on the linker length. For each additional ([E/L]PPPP)-segment the Rg increased by ~0.4–0.5 Å (Fig. 2c). Linker P5, which consists of 25 residues forms an exception to this as it exhibited an Rg similar to that of the P4 linker, i.e. ~1.8 Å (Fig. 2c). Overall bending of the long P-linkers may have contributed to this occurrence (Fig. 2a).

### Influence of auxiliary YkuN on CYP109B1 catalysis driven by the YkuN-Fpr fusion constructs

**Figure 3:**
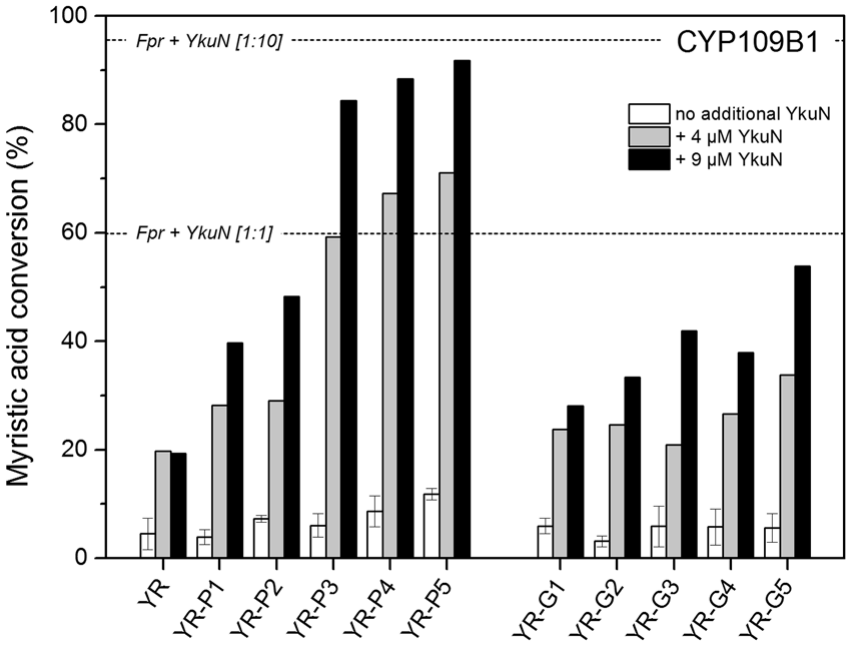
Influence of auxiliary YkuN on CYP109B1 catalysis driven by the different YkuN-Fpr fusion constructs. Myristic acid conversion with CYP109B1 (1 µM) was carried out in the presence of the different Ykun-Fpr fusion constructs either in the absence (- YkuN) or presence of additional YkuN (4 or 9 µM). In all cases reactions were started by the addition of a mixture of NADPH and myristic acid and allowed to proceed for 120 min under the support of an NADPH regenerating system. The Fpr/YkuN/CYP109B1 system reconstituted at 1:10:1 and 1:1:1 ratio, respectively achieved 96% and 60% conversion of myristic acid in 120 min.

In comparison to the separate redox enzymes the fusion constructs exhibit a reduced performance at low protein concentration (Fig. 1b), which may indicate that one (or both) of the fused redox partners is limiting the P450-catalysed reaction. With reconstituted P450 systems the electron shuttle ferredoxin/flavodoxin is usually a major limiting factor that is typically overcome by applying an excess of this electron transfer protein^16, 28, 29^. To investigate this, the different fusion constructs (1 µM) were incubated together with CYP109B1 (1 µM) in the absence or presence of an excess of YkuN (4 or 9 µM). In all cases, mixing the fusion proteins with additional YkuN led to an increase in myristic acid conversion (Fig. 3), which confirms that YkuN is limiting the reaction. By increasing the concentration of the fusion constructs itself in the P450-catalysed reaction, this apparent limitation was also (partly) overcome (Fig. 1b).

Since the reductase (Fpr) domain of the fusion constructs is capable of donating electrons to the auxiliary electron shuttle YkuN, this also indicates that no strict functional coupling between the Fpr and YkuN domain exists within the fusion constructs. Generally, electron transfer rates are thought to be controlled in part by the distance between the redox centres^33^. Considering the YkuN-Fpr fusion constructs it is likely that both the length and the structural properties of the linker contribute to the relative distance between the respective redox centres. Regarding the rigid nature of the ([E/L]PPPP)_n_ linkers (Fig. 2) it seems plausible that in particular long P-linkers might facilitate a higher degree of separation of the fusion partners. In line with this, the increase in conversion upon YkuN addition was much more pronounced for fusion constructs carrying the rigid P-linkers (Fig. 3), which more effectively maintain an extended conformation than their glycine-rich counterparts (Fig. 2). In fact, the highest increase in myristic acid conversion governed by the addition of YkuN was observed for the YR-P5 construct which carries the longest P-linker tested (25 residues), achieving a similar conversion as the comparable 1:10 system of non-fused Fpr/YkuN (Fig. 3).

### NADPH oxidation rates and coupling efficiencies measured with the YkuN-Fpr fusion enzymes

The overall performance of a reconstituted P450-redox partner system depends amongst others on its NAD(P)H oxidation rate and the coupling efficiency between NAD(P)H oxidation and P450 substrate conversion. Since delivery of electrons to CYP109B1 depends on functional reductase (Fpr) as well as flavodoxin (YkuN), a loss of electrons may occur either upon electron transfer from Fpr to YkuN or from YkuN to CYP109B1, which in both cases will lead to uncoupling between NADPH oxidation and product formation.

The NADPH oxidation rate and the coupling efficiency were determined for various reconstituted systems (Table 1). CYP109B1 reconstituted with the non-fused redox partners exhibited the highest NADPH oxidation rate, i.e. 26 nmol·(nmol P450)^−1^·min^−1^, along with a coupling efficiency of 28.3%. In contrast, for the fusion constructs substantially lower NADPH consumption rates were observed, whereas the coupling efficiency was markedly improved (Table 1). Direct attachment of YkuN to Fpr (YR) led to a ~1.8-fold improvement of the coupling efficiency (49.8%) and a more than 8-fold decrease in NADPH oxidation rate (Table 1). In comparison to YR, the superior construct YR-P5 (Fig. 1) showed a similar coupling (48.9%), whereas the NADPH oxidation rate was 3.6-fold higher, i.e. 11.3 nmol·(nmol CYP)^−1^·min^−1^ (Table 1). The highest coupling efficiency of 81.2% was however observed for YR-G5, which, notably, exhibited the lowest NADPH oxidation rate of 1.4 nmol·(nmol P450)^−1^·min^−1^.

**Table 1:** NADPH oxidation rate and coupling efficiency of the CYP109B1-catalysed conversion of myristic acid, supported by different redox partners.

Reconstituted system^a^	NADPH oxidation rate^b^	Coupling^c^ efficiency (%)
Fpr / YkuN / CYP109B1 – [4:4:1]	26.0 ± 1.9	28.3 ± 3.6
YR / CYP109B1 – [4:1]	3.1 ± 0.3	49.8 ± 8.3
YR-P1 / CYP109B1 – [4:1]	12.7 ± 1.3	62.1 ± 5.1
YR-P5 / CYP109B1 – [4:1]	11.3 ± 1.9	48.9 ± 3.4
YR-G1 / CYP109B1 – [4:1]	6.6 ± 0.3	72.4 ± 3.6
YR-G5 / CYP109B1 – [4:1]	1.4 ± 0.2	81.2 ± 4.1

Presented data represent average values of at least three independent reactions. The ratio of NADPH:myristic acid employed was 1:1 (200 µM each, respectively) ^a^Values in brackets indicate applied ratio as well as final concentration (µM) of indicated proteins. ^b^Rates are given in nmol NADPH per nmol CYP109B1 per minute. The background NADPH oxidation rate in the absence of redox partner(s) was 0.1 ± 0.0. ^c^The coupling efficiency equals the myristic acid conversion achieved upon NADPH depletion (see also Supplementary Fig S5).

### Reduction of CYP109B1 Fe^3+^-heme by the YkuN-Fpr fusion constructs

Since the YkuN-Fpr fusion constructs exhibited attenuated NADPH oxidation rates along with improved coupling, the question arises whether the crucial step of electron transfer to the P450 is also influenced upon attachment of YkuN to Fpr. The reduction of the Fe^3+^ to Fe^2+^-heme can be conveniently monitored by the addition of carbon monoxide (CO), yielding a stable complex that exhibits the characteristic absorbance maximum at 450 nm^34^.

Indeed, absorbance spectroscopy of the anaerobic reduction of CYP109B1 in the presence of CO by the different redox partners revealed the formation of the typical absorbance peak at ∼450 nm, which is governed by the reduction of Fe^3+^ to Fe^2+^-heme and the subsequent formation of the stable heme-Fe^2+^-CO complex. Typical traces for the time dependent reduction are shown in Supplementary Fig. S4. P450 reduction occurred rather slowly under the tested conditions (Fig. S4). Data were fit to a bi-exponential function, revealing a slow and a fast phase; the corresponding reduction rates are shown in Table 2. Reduction occurred fastest by the non-fused Fpr/YkuN, exhibiting a *k*_1_ of 0.009 s^−1^ and *k*_2_ of 0.078 s^−1^. For the linker-less fusion YR corresponding rates were 4.5 and 2.6-fold decreased. In contrast, for the fusion constructs carrying the long P5 or G5 linker, *k*_1_ was virtually identical to that of the non-fused Fpr/YkuN and *k*_2_ was decreased to a lesser extent (Table 2). Of the tested fusion constructs, the superior YR-P5 also exhibited the highest heme-iron reduction rate (*k*_1_ 0.008 s^−1^ and *k*_2_ 0.045 s^−1^). Thus, introduction of the P5 linker (25 residues) between the fused YkuN and Fpr led to a substantial improvement of the heme-iron reduction rate.

**Table 2:** Reduction of CYP109B1 Fe^3+^-heme by different redox partners.

Reconstituted system^a^	Heme-iron reduction rate^b^	
	*k*_1_ (s^−1^)	*k*_2_ (s^−1^)
Fpr / FldA / CYP109B1 – [4:4:1]	0.004 ± 0.001	0.027 ± 0.008
Fpr / YkuN / CYP109B1 – [4:4:1]	0.009 ± 0.001	0.078 ± 0.023
YR / CYP109B1 – [4:1]	0.002 ± 0.001	0.030 ± 0.002
YR-P5 / CYP109B1 – [4:1]	0.008 ± 0.002	0.045 ± 0.005
YR-G5 / CYP109B1 – [4:1]	0.007 ± 0.001	0.035 ± 0.002

^a^Values in brackets indicate applied ratio as well as final concentration (µM) of the proteins in the employed reconstituted systems. ^b^The heme-iron reduction rates were measured as described in the Methods section. Kinetic traces were fit to a bi-exponential function, revealing a slow and a fast phase. Typical kinetic traces and corresponding fits are shown in the online Supplementary Information. Presented reduction rates represent average values of at least three independent reactions carried out under anaerobic conditions at 20 °C.

### Versatility of the YkuN-Fpr redox fusion enzymes

From a biotechnological point of view, a redox partner system desirably should have the ability to transfer electrons to different terminal acceptors. To demonstrate cross-reactivity, selected fusion constructs were additionally tested with CYP154E1 from *Thermobifida fusca* YX, CYP106A2 from *Bacillus megaterium* ATCC 13368 and bovine CYP21A2.

**Figure 4:**
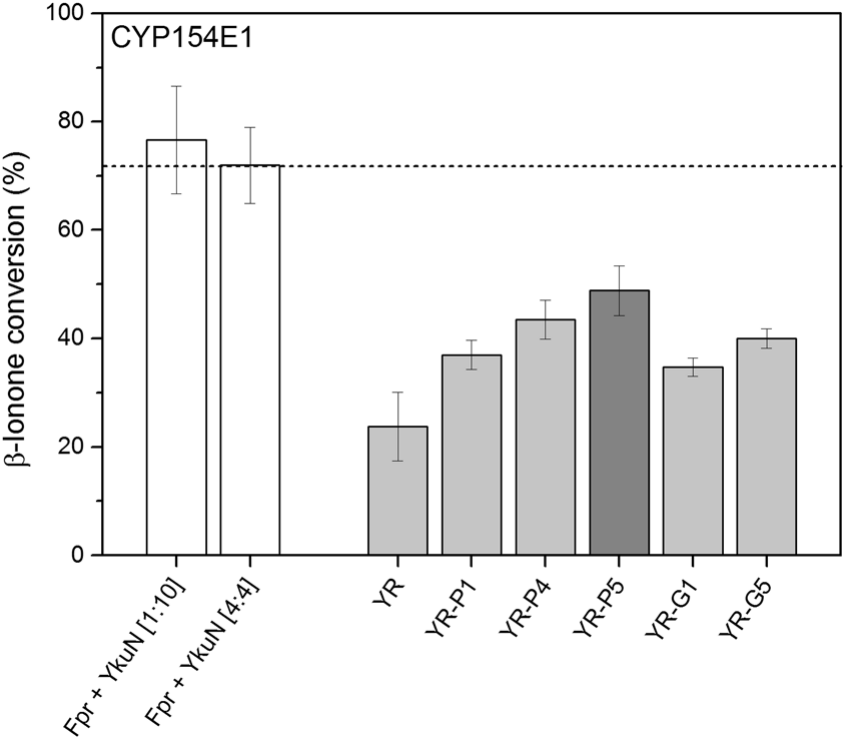
Conversion of β-ionone by *T. fusca* YX CYP154E1 supported by selected fusion constructs. Reactions were carried out in the presence of 1 µM CYP154E1 and 4 µM of indicated fusion constructs. Control reactions with non-fused Fpr/YkuN were conducted with 1 µM CYP154E1 together with either 1 µM Fpr + 10 µM YkuN [1:10], or 4 µM Fpr + 4 µM YkuN [4:4]. In all cases the initial concentration of the substrate β-ionone was 2 mM. Reactions were started by the addition of NADP^+^ under support of an NADPH regenerating system and stopped after 1 h.

CYP154E1 is a versatile monooxygenase that converts a large variety of substrates, including fatty acids and alcohols, as well as acyclic and bulky cyclic terpenoids^17, 35, 36^. Since Fpr/YkuN are known to effectively support CYP154E1 catalysis^17, 35, 36^ it is expected that the YkuN-Fpr fusion constructs are also able to act as surrogate redox partners. To investigate this, selected fusion constructs were tested for their ability to support CYP154E1 catalysis using β-ionone as substrate (Fig. 4). Ionones are cyclic terpenoids that are key fragrance components used for the production of perfumes, cosmetics and other fine chemicals^37^. Moreover, β-ionone is an important intermediate in the manufacturing of vitamin A, while its oxygenated derivative 4-hydroxy-β-ionone is a key intermediate in the synthesis of carotenoids and the plant hormone abscisic acid^38–40^.

Non-fused Fpr/YkuN (1:10 or 4:4 system) supported CYP154E1 catalysis, achieving >70% conversion of β-ionone in 1 h (Fig. 4). Thus, in addition to the previously reported terpenoid substrates^36^, β-ionone is also converted by CYP154E1. As expected, the fusion constructs were able to support CYP154E1 catalysis, of which YR-P5 again proved most effective at driving P450 catalysis (Fig. 4). In the presence of YR-P5 ~1 mM of β-ionone was converted in 1 h, yielding a turnover of ~16 min^−1^, while non-fused Fpr/YkuN (4:4) supported a turnover of ~24 min^−1^. The YR-P5/CYP154E1 (4:1) system achieved a coupling efficiency of ~50%, whereas with the non-fused enzymes ~44% was measured (Supplementary Table S4). Furthermore, under cofactor regeneration conditions, total turnover numbers (TTN) were 1,582 with YR-P5 and 1,948 with the non-fused Fpr/YkuN (Supplementary Table S5). Regardless whether Fpr and YkuN were fused or not, in all cases a major oxidation product was formed, which was identified as 4-hydroxy-β-ionone by comparison to MS analysis of an authentic reference substance^41^.

To further substantiate functional promiscuity, the best construct YR-P5 was tested with CYP106A2 and CYP21A2 using progesterone as substrate in both cases. It is of note that these P450s have not been tested previously with Fpr/YkuN as surrogate redox partners. CYP106A2 is a regio- and stereoselective 15β-hydroxylase of 3-oxo-∆^4^-steroids^42, 43^ that recently has been demonstrated to also accept 3-hydroxy-∆^5^-steroids as well as di- and triterpenes as substrates^44, 45^. Herein, the bovine adrenodoxin reductase (AdR) and adrenodoxin (Adx) typically serve as surrogate redox partners. Interestingly Fpr/YkuN and its fused derivative YR-P5 were able to functionally substitute for AdR/Adx in the CYP106A2-mediated conversion of progesterone (Table 3). Moreover, performance of the 4:4:1 Fpr/YkuN/CYP106A2 system was virtually identical to that of the 4:4:1 AdR/Adx/CYP106A2 system, both with respect to progesterone conversion and product distribution (Table 3). With both systems nearly all progesterone was converted after 120 min and 15β-hydroxyprogesterone was the main product formed (Table 3). The fusion construct YR-P5 also supported CYP106A2 catalysis, albeit with lower efficacy (Table 3). The YR-P5/CYP106A2 (4:1) system achieved ~46% conversion after 120 min, producing almost exclusively 15β-OH-progesterone (Table 3

**Table 3:** Product distribution for the CYP106A2-catalysed conversion of progesterone supported by different redox partners.

Redox partner(s)	Ratio^a^	Conversion time (min)	Progesterone conversion (%)	Compounds (%)	15β-OH^b^	mono-OH^b^	poly-OH^b^
				Prog.^b^			
AdR/Adx	(4:4:1)	30	96.0 ± 0.5	4.0 ± 0.5	81.7 ± 1.0	8.7 ± 1.3	5.6 ± 0.9
		120	99.0 ± 0.1	1.0 ± 0.1	81.1 ± 0.5	6.9 ± 0.5	11.0 ± 0.7
	(10:10:1)	30	96.0 ± 0.6	4.0 ± 0.6	81.5 ± 0.5	7.9 ± 0.1	6.6 ± 0.2
		120	96.7 ± 1.0	3.3 ± 1.0	82.6 ± 1.6	7.9 ± 0.3	6.1 ± 1.4
Fpr/YkuN	(4:4:1)	30	92.7 ± 0.7	7.3 ± 0.7	80.4 ± 0.5	4.0 ± 0.4	8.3 ± 0.1
		120	98.9 ± 0.0	1.1 ± 0.0	81.9 ± 0.0	4.3 ± 0.0	12.8 ± 0.0
	(10:10:1)	5 (500 µM)^c^	84.7 ± 5.4	15.3 ± 5.4	80.4 ± 3.1	2.0 ± 0.2	2.2 ± 2.2
		30	91.5 ± 1.3	8.5 ± 1.3	63.0 ± 1.0	4.4 ± 0.1	24.1 ± 0.2
		60	94.0 ± 0.1	6.0 ± 0.1	58.4 ± 0.7	5.2 ± 0.7	30.5 ± 0.1
		90	98.5 ± 0.4	1.5 ± 0.4	68.2 ± 4.2	6.5 ± 0.4	23.8 ± 4.7
		120	97.8 ± 0.2	2.1 ± 0.2	66.3 ± 2.6	6.4 ± 0.4	25.2 ± 3.0
YR-P5	(4:1)	30	19.5 ± 2.6	80.5 ± 2.6	19.1 ± 2.1	0.4 ± 0.5	—
		60	29.6 ± 5.4	70.4 ± 5.4	29.2 ± 4.9	0.4 ± 0.6	—
		90	39.7 ± 16.2	60.3 ± 16.2	39.7 ± 16.2	—	—
		120	45.9 ± 15.2	54.1 ± 15.2	44.0 ± 13.4	1.9 ± 1.9	—
	(10:1)	5 (500 µM)^c^	29.7 ± 0.6	70.3 ± 0.6	29.7 ± 0.6	—	—
		30	75.9 ± 3.8	24.1 ± 3.8	71.3 ± 4.2	3.4 ± 0.5	1.2 ± 0.9
		60	80.0 ± 0.9	20.0 ± 0.9	74.9 ± 1.4	3.2 ± 0.8	1.8 ± 0.3
		90	90.7 ± 5.3	9.3 ± 5.3	81.5 ± 3.7	3.6 ± 0.8	5.6 ± 1.0
		120	94.9 ± 0.7	5.1 ± 0.7	84.9 ± 0.7	4.0 ± 1.3	6.1 ± 0.2

Data represent average values of three independent reactions (using 200 µM progesterone) with indicated standard deviation. ^a^Redox partner-CYP106A2 ratio of reconstituted system. ^b^ *B. megaterium* CYP106A2 hydroxylates progesterone at positions 15β, 6β, 11α and 9α^76^; mono-OH, monohydroxylated progesterone at positions other than 15β; poly-OH, di- or polyhydroxylated progesterone. ^c^To assess turnover, conversion reactions were carried out for 5 min using 500 µM progesterone.

). By increasing the YR-P5 concentration (10:1 system) conversion could be enhanced to near completion (95%) after 120 min, while only minor amounts of undesired polyhydroxylated progesterone were formed (up to 6%).

To assess turnover rates, reactions were carried out under conditions in which essentially no overoxidation products were formed (5 min reactions using 500 µM progesterone). With the Fpr/YkuN/CYP106A2 system (10:10:1) nearly 85% of the progesterone was converted, thus achieving a turnover of 169 min^−1^, whereas with the YR-P5/CYP106A2 system (10:1) nearly 30% progesterone conversion was achieved, thus yielding a turnover of 59.4 min^−1^.

**Figure 5:**
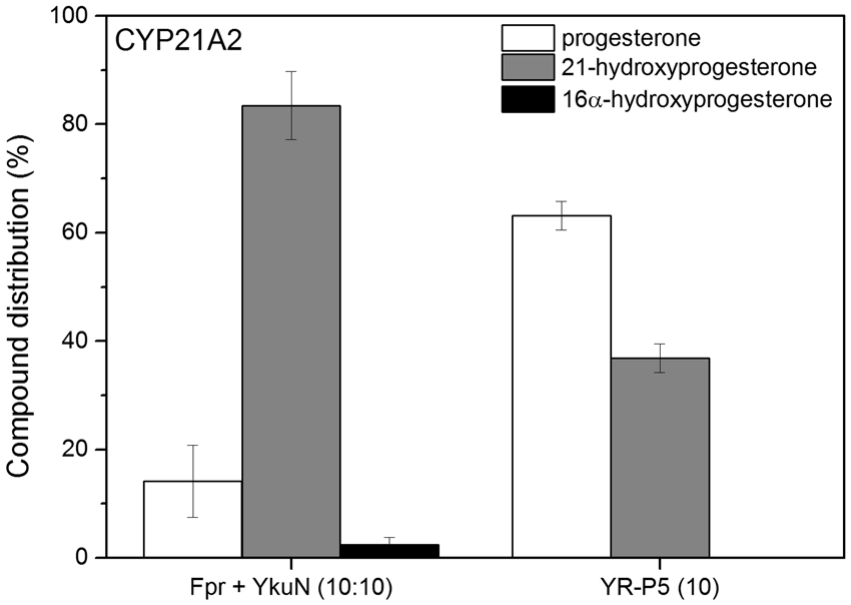
Conversion of progesterone by bovine CYP21A2 supported by Fpr/YkuN and their fused derivative YR-P5. In all cases reactions were carried out in the presence of 0.5 µM CYP21A2, 200 µM progesterone and 5 µM of indicated redox partner(s). Corresponding ratios of redox partner(s) relative to CYP21A2 are indicated in brackets. Reactions were started by the addition of NADPH and supported by a NADPH regenerating system. Reactions were stopped after 30 min.

To further illustrate versatility of YR-P5, bovine CYP21A2, which is a membrane-bound microsomal P450, was used as terminal electron acceptor. CYP21A2 is involved in the biosynthesis of steroid hormones, and typically catalyses the hydroxylation of the carbon atom 21 in steroids^46^. In general, CYP21A2 obtains the necessary electrons from NADPH via an FAD and FMN-containing microsomal CPR^8^. However, it is also able to use AdR and Adx as redox partners^47^. Notably, YR-P5 supported CYP21A2 catalysis; with YR-P5 40% of the (200 µM) progesterone was converted to 21-hydroxyprogesterone in 30 min, while ~80% conversion was achieved with non-fused Fpr/YkuN (Fig. 5). Taken together, the Fpr/YkuN redox pair and its fused derivative YR-P5 are able to support the activity of the steroidogenic CYP106A2 and CYP21A2 *in vitro*, which indicates that these bacterial electron transfer systems may serve as alternatives to the mammalian electron transfer systems AdR/Adx and CPR.

## Discussion

Flavodoxins are promiscuous electron carriers that donate electrons to structurally and functionally diverse enzymes, including pyruvate-formate lyase^48^, ribonucleotide reductase^49^, key enzymes in photosynthesis^50^, nitrogen fixation^51^, methionine^52^ and biotin^53^ synthesis, but also P450s^16, 27, 29, 54, 55^. The mixed redox pair Fpr/YkuN outperformed the physiological redox pair Fpr/FldA from *E. coli* in supporting CYP109B1 catalysis (Fig. 1a), which suggests that electron transfer by the flavodoxin YkuN is more effective. Since YkuN and CYP109B1 both originate from *B. subtilis* it is possible that YkuN is the physiological redox partner of CYP109B1 and therefore a more favourable electron carrier than the heterologous FldA.

Typically, the midpoint potentials of the oxidised/semiquinone (E_1_′) and semiquinone/hydroquinone (E_2_′) couples reported for the short-chain flavodoxin YkuN are higher than those for the long-chain flavodoxin FldA, i.e. −105 mV and −382 mV^27^ *vs*. −254 mV and −433 mV, respectively^55^. E_1_′ and E_2_′ of the employed *E. coli* reductase Fpr are −308 mV and −268 mV, respectively^55^. Indeed, the anaerobic reduction of CYP109B1 heme iron by YkuN occurred 2–3 times faster than by FldA (Table 2). Similarly, stopped-flow experiments monitoring the reduction of *B. subtilis* CYP107H1 have indicated that YkuN is more effective than FldA in first electron transfer to palmitoleate-bound CYP107H1, as evidenced by a ~12-fold higher *k*_red_^27^. Thus, accelerated electron transfer likely contributed to the overall better performance of YkuN compared to FldA in the CYP109B1-catalysed conversion of myristic acid (Table 2, Fig. 1a).

The inherent dependence of P450s on electron transfer proteins presents a challenging limitation in their biotechnological exploitation. To simplify redox chains and to improve the catalytic properties of P450 systems, a variety of man-made P450 fusion enzymes have been successfully created using a variety of molecular approaches, including “Molecular Lego”^22^, “LICRED”^56^, and”PUPPET”^57^. Here, the versatility and superior properties of YkuN were exploited to generate redox fusion enzymes capable of driving catalysis of different CYPs, using the previously established DuaLinX procedure^19^ for linker engineering. The catalytic performance of a reconstituted P450 system is often limited by a low coupling efficiency. Uncoupling events waste expensive reduced cofactors (NAD(P)H) and lead to the generation of reactive oxygen species that can cause enzyme inactivation^58^. With non-physiological redox chains the coupling efficiency is often particularly poor (less than 20%)^16^ and also when P450s catalyse reactions with non-physiological substrates, coupling efficiencies are frequently severely diminished (<10%)^58^. Achieving a high coupling efficiency is thus a particularly challenging task.

Direct attachment of YkuN to Fpr via genetic fusion led to a 1.8-fold improvement of the coupling efficiency in CYP109B1-catalysed reactions (Table 1). Coupling efficiency could be even further improved by insertion of an appropriate linker between the fusion partners. In case of the YR-G5 construct a coupling efficiency as high as 81.2% was achieved. Notably, in all cases the CYP109B1 systems reconstituted with the different YkuN-Fpr fusion constructs exhibited a higher coupling efficiency than those reported previously using a variety of different redox partners (1.8–45.8%)^16^, which is an advantage under cofactor regeneration condition. However, the NADPH oxidation rate was markedly reduced.

Despite improved coupling, overall performance of the parental linker-less YkuN-Fpr fusion construct in P450-catalsed reactions was rather modest in comparison to the non-fused enzymes (Figs 1 and 4). A > 8-fold reduction in NADPH oxidation rate (Table 1) combined with slower electron transfer to the CYP109B1 (Table 2) likely contributed to the reduced performance of the YR fusion construct. Insertion of a suitable linker between the fusion partners however, substantially improved the overall performance. Herein, long ([E/L]PPPP)_n_ linkers (n = 4–5) were particularly effective (Figs 1 and 4), which is consistent with previous findings for fusions between *E. coli* FldA and Fpr^19^. At protein concentrations ≥4 µM, the superior construct YR-P5 acted as a nearly equivalent substitute for non-fused Fpr/YkuN in driving CYP109B1 catalysis (Fig. 1). Insertion of the P5 linker between the fused YkuN and Fpr resulted in a 1.5-fold increase in heme iron reduction rate (Table 2, *k*_2_) along with a 3.6-fold higher NADPH oxidation rate, while maintaining a high coupling efficiency of nearly 50% (Table 1), thus contributing to the improved overall performance (Fig. 1). However, despite poorer coupling efficiency, the non-fused redox partners proved generally more effective than YR-P5 in supporting P450 catalysis (Table 3, Figs 1, 4 and 5), which is likely governed by faster overall electron transfer (accelerated NADPH oxidation as well as P450 reduction). The higher amount of progesterone overoxidation products formed by CYP106A2 when the non-fused redox partners are used as opposed to YR-P5, is also consistent with this notion (Table 3).

Examination of the linker properties using MD simulations revealed that the (GGGGS)_n_ linkers are highly flexible and tend to adopt compact random structures, whereas the ([E/L]PPPP)_n_ linkers maintain their linear conformation and are therefore structurally more rigid (Fig. 3). These results are consistent with the notion that glycine-rich and proline-rich amino acid sequences are flexible and rigid in nature, respectively^59–61^. Moreover, the high mobility of glycine-rich linkers is apparent from their lack of structural resolution in X-ray structures of a multitude of artificial fusion proteins^62^. Due to their flexibility, glycine-rich linkers are unstructured and tend to provide limited domain separation^63, 64^, whereas structurally rigid linkers, such as proline-rich linkers are more likely to separate the fusion partners^60, 65^.

For flavodoxins the binding areas for the electron donating reductase and the terminal electron acceptor (P450) are partially overlapping, which precludes the formation of a ternary protein complex^66, 67^. Considering the fusion constructs, an optimal linker should therefore facilitate the formation of an electron transfer complex between Fpr and YkuN as well as between YkuN and the P450. In view of the superior performance of the YR-P5 construct these criteria are apparently best met by the ([E/L]PPPP)_5_ linker. Indeed, both the activity of Fpr (NADPH oxidation rate) and YkuN (heme-iron reduction) are reasonably well preserved in the YR-P5 construct (Tables 1 and 2). Herein, the rigid P5 linker may restrict the degrees of freedom of the fused redox partners, while allowing them to mutually interact in an effective manner, thus promoting *intra*molecular electron transfer. On the other hand, the extended rigid P5 linker (25 residues) may increase the distance between the redox centres of the fusion partners, thereby disfavouring *intra*molecular electron transfer and facilitating electron transfer between fusion proteins (*i.e. inter*molecularly). The available experimental data for YR-P5 seems to be more consistent with a predominantly *inter*molecular electron transfer pathway. YR-P5 lacks a strict functional coupling between the Fpr and YkuN domain and its Fpr domain is readily accessible for external YkuN (Fig. 3), which is likely facilitated by a higher degree of separation of the fusion partners in case of the P5 linker. Moreover, P450 mediated catalysis exhibited a higher order (sigmoidal) dependence on fusion enzyme concentration suggesting an *inter*molecular contribution, whereas a linear dependence would have been indicative of exclusive *intr*amolecular electron transfer (Fig. 1b). At elevated redox partner concentration, which increases the collision frequency between fusion proteins, apparent limitations in electron transfer were (partially) overcome and the performance of YR-P5 was similar to that of the non-fused Fpr/YkuN (Fig. 1b).

Finally, the superior construct YR-P5 exhibits versatility as it effectively supported monooxygenase activity of functionally diverse P450s, including *B. subtilis* CYP109B1 (Fig. 1), *T. fusca* YX CYP154E1 (Fig. 4), *B. megaterium* CYP106A2 (Table 3) and bovine CYP21A2 (Fig. 5). Thus, functional promiscuity of YR-P5 is not limited to bacterial P450s.

Considering the feasibility of the Fpr/YkuN system and its fused derivative YR-P5 for applied biocatalysis, the TTN presented here are rather modest. In case of CYP154E1 with β-ionone as substrate TTN were 1,582 with YR-P5 and 1,948 with the non-fused Fpr/YkuN (Supplementary Table S5). On the other hand, we recently observed that a Fpr/YkuN/CYP154E1 system can achieve TTN of up to 20,000 with stilbene as substrate^68^. Although TTN in P450-based multi-component systems depend on many factors, the P450-substrate match seems to play a prominent role. For example, CYP154C5 catalysis supported by PdR/Pdx achieved TTN of 2,440 with progesterone and 3,341 with androstenedione under optimized conditions^69^. For the natural fusion P450 BM3 from *Bacillus megaterium* (and mutants thereof), TTN values range from 890 with omeprazole^70^, 2,200 with *n*-octane^71^, to 6,195 with anisole^72^, and even TTN as high as 24,363 or 45,000 have been reported in case of cyclooctane^73^ and propane^74^, respectively.

Taken together, the intrinsic dependence on redox partner(s) represents an important limiting factor in the utilization of P450s as biocatalysts. Availability of suitable redox partner(s) is therefore a prerequisite to successfully explore the catalytic potential of P450s^10^. By covalent fusion the complexity of the bacterial Fpr/YkuN electron transfer chain was effectively reduced, allowing easy protein production, purification and handling. Moreover, a stabilising effect on Fpr was noted upon fusion to YkuN (Supplementary Fig. S2). Through linker engineering the activity of the fusion construct could be tuned such that the high activity of the individual redox partners and the promiscuity for different P450s was largely preserved, with overall best performance of the YR-P5 construct. The versatile YR-P5 may serve as an effective surrogate electron transfer system for exploitation of the catalytic potential of (orphan) P450s.

## Methods

### Generation of YkuN-Fpr fusion constructs

Construction of the *ykuN:fpr* fusion gene was performed by fusing the *Bacillus subtilis* flavodoxin gene *ykuN* lacking its stop codon to the 5’-end of the flavodoxin reductase gene *fpr* from *E. coli* JM109 by means of PCR with overlapping primers using pET16b-*ykuN* ^16^ and pET11a-*hAR* ^19^ as respective DNA-templates. Three extra nucleotides (GCC) were incorporated at the fusion site to create a unique *Nco*I restriction site that facilitates linker (DuaLinX) insertion. DuaLinX are double stranded DNA elements of different lengths with *Nco*I-compatible overhangs, which depending on their orientation after insertion code for either (GGGGS)_n_ or ([E/L]PPPP)_n_ linkers (n = 1–5)^19^. Moreover, molecular dynamics (MD) simulations were carried out on these peptide linkers to gain insight in their intrinsic structural properties. Procedures for fusion PCR, linker insertion and MD simulations are described in detail in the accompanying online Supplementary Information. The different YkuN-Fpr fusion proteins were produced in *E. coli* BL21(DE3) and purified from the cytosol via their N-terminal His_6_-tag. Expression and purification procedures of the various proteins used in this study are described in the online Supplementary Information.

### P450-catalysed conversion reactions

CYP109B1 reaction mixtures contained 50 mM Tris-HCl pH 7.5, 1 mM MgCl_2_, 4 mM glucose-6-phosphate (G6P), 1 U G6P-dehydrogenase from *Saccharomyces cerevisiae*, 1 µM CYP109B1 and redox protein(s) as indicated. Stock solutions of myristic acid (10 mM, dissolved in DMSO) and NADPH (1 mM, dissolved in 50 mM Tris-HCl, pH 7.5) were pre-mixed at 1:10 ratio, respectively. Hereof, 44 µl was used to start the reactions (final concentrations: 200 µM for both myristic acid and NADPH, and 2% (v/v) DMSO).Total reaction volume was 200 µl. Reactions were allowed to proceed for 120 min at 30 °C and 300 rpm in a thermo-shaker and then stopped by the addition of HCl (8 µl, 37% w/w). For quantitative gas-liquid chromatography/mass spectrometry (GC/MS) analysis, the internal standard tridecanoic acid (dissolved in 100% DMSO) was added to a final concentration of 50 μM. For determination of NADPH consumption rates and coupling efficiencies, the CYP109B1 reactions mixtures lacked G6P-dehydrogenase and were transferred to 96-microwell plate followed by 2 min incubation at 30 °C in a TECAN infinite m200 pro plate reader equipped with an injector module. Reactions were started by injecting 44 µl of the NADPH-myristic acid solution (described above) and the change in absorbance was measured at 340 nm at 30 °C. The NADPH consumption rate was calculated using ε_340_ = 6.22 mM^−1^cm^−1^. After all NADPH was consumed, HCl and internal standard were added as described above for GC/MS analysis. The employed ratio of NADPH:myristic acid was 1:1 (200 µM each, respectively) and thus the coupling efficiency equals the proportion (%) of myristic acid converted upon NADPH depletion. CYP154E1 activity was determined in 125 µl reaction mixtures containing 100 mM KPi pH 7.5, 1 µM CYP154E1 together with 4 µM each of Fpr and YkuN (unless stated otherwise) or 4 µM of YkuN-Fpr fusion construct, 2 mM β-ionone (dissolved in DMSO, yielding a final DMSO concentration of 2% v/v in the conversion assay) and 200 µM NADP^+^. For NADPH regeneration and removal of hydrogen peroxide, 0.63 U GDH and 20 mM glucose, and 75 U catalase from bovine liver (Sigma-Aldrich) were also included, respectively. Reactions were performed at 30 °C and 600 rpm for 1 h and then extracted by the addition of 100 µl ethyl acetate under vigorous vortexing. For quantification purposes α-ionone (internal standard) was added to a final concentration of 2 mM. CYP106A2 reactions were performed in 250 µl total reaction volume and contained 50 mM KP_i_ pH 7.4, 0.5 µM CYP106A2 together with the individual or fused redox partners at 2 or 5 µM, 200 µM of the substrate progesterone (dissolved in 100% ethanol), 5 mM G6P, 1 U G6P-dehydrogenase and 1 mM MgCl_2_.The reactions were started by addition of NADPH (final concentration 100 µM) and incubated at 30 °C and 900 rpm. At different time points, the reactions were stopped by the addition of ethyl acetate. Extraction was carried out two times using 250 µl ethyl acetate each, by vigorous mixing. CYP21A2 reactions (250 µl) contained 50 mM HEPES pH 7.4, 0.05% (v/v) Tween 20, 0.5 µM CYP21A2, 5 µM redox partner, 200 µM of the substrate progesterone (dissolved in 100% ethanol), 5 mM G6P, 1 U G6P-dehydrogenase and 1 mM MgCl_2_.The reactions were started by addition of NADPH (final concentration 100 µM) and allowed to proceed for 30 min at 37 °C and 900 rpm. Extraction and HPLC analysis was as described for CYP106A2.

### Quantification of substrates and product identification

Presented conversion data are average values of multiple (at least three) independent conversion reactions with indicated standard deviations. Quantification of substrates and product identification were carried out as follows. CYP109B1 **-** For quantification of myristic acid (MA), the detector response was calibrated using tridecanoic acid (TDA) as internal standard. For this purpose, TDA at a fixed concentration of 50 µM was mixed with MA at final concentrations ranging from 5–200 μM in 50 mM Tris-HCl buffer, pH 7.5. Samples were then treated, extracted and analysed by GC/MS as described for normal conversion reactions. The ratio of the area of the MA to that of the TDA was plotted against the MA concentration and yielded a linear calibration curve. CYP154E1 - For the quantification of β-ionone, a calibration curve was recorded with different concentrations of β-ionone ranging from 0.05–5 mM using 2 mM of α-ionone as internal standard. Conversion products were identified by their characteristic mass fragmentation patterns. In case of β-ionone conversion, the main conversion product 4-hydroxy-β-ionone was identified by its characteristic mass fragmentation pattern and retention time, and compared to those of the authentic reference compound. CYP106A2/CYP21A2 – The organic phases were combined and evaporated, after which the samples were stored at −20 °C until reversed-phase HPLC analysis. Evaluation of progesterone conversion and product formation was performed by HPLC peak integration, with the total peak area of substrate plus products set to 100%. The procedures for GC/MS and HPLC analyses are described in detail in the accompanying online Supplementary Information.

### Reduction of the heme-iron of CYP109B1 by various redox partners

Reduction of the heme iron was monitored by using a CO-difference spectroscopy-based method^34^. The reduction rate of heme Fe^3+^ to Fe^2+^ was measured under anaerobic conditions (nitrogen atmosphere) in a glovebox (Glovebox Systemtechnik, Malsch, Germany) at room temperature (20 °C). Buffer and protein solutions were incubated under anaerobic conditions to obtain oxygen-free solutions. Reduction of the P450 can be determined in the presence of CO by monitoring the formation of the characteristic absorbance peak at 450 nm. Absorbance spectra (340–700 nm) were recorded using a diode array spectrophotometer (TIDAS E BASE / VIS-NIR, J&M Analytik, Essingen, Germany). To this end, CYP109B1 (2 µM) and myristic acid (400 µM) were premixed in buffer containing 50 mM Tris-HCl, pH 7.5 and flushed with 1 ml of CO gas using a gas-tight syringe. Redox partner(s) were premixed in a separate solution containing 50 mM Tris-HCl, 8 µM redox partner(s) and 2 mM NADPH. Then, 100 µl of the P450 solution were transferred to UV-cuvette and manually mixed with 100 µl of the solution containing the reduced redox partner(s). Kinetic traces were extracted from the acquired spectra by plotting the A_450_ −A_490_ against the time^75^. Kinetic data were analysed using OriginPro 9.0 software using a fitting routine with two-exponential steps. The equation used for the calculations was A = A_1_(1 − ${{\rm{e}}}^{{{\rm{k}}}_{1}{\rm{t}}}$) + A_2_(1 − ${{\rm{e}}}^{{{\rm{k}}}_{2}{\rm{t}}}$) + C.

### Data availability

The data generated or analysed during the current study are either included in this published article (and its Supplementary Information files) or are available from the corresponding author on reasonable request.

## Electronic supplementary material

SUPPLEMENTARY INFO
