# Supplementary material for: Engineering of versatile redox partner fusions that support monooxygenase activity of functionally diverse cytochrome P450s
Source: Sci Rep. 2017 Aug 29;7:9570. doi: 10.1038/s41598-017-10075-w (PMC5575160; doi:10.1038/s41598-017-10075-w)
Supplement: Supplementary file 1 — SUPPLEMENTARY INFO [file 41598_2017_10075_MOESM1_ESM.pdf]

Supplementary Information

to

Engineering of versatile redox partner fusion enzymes that support monooxygenase activity of functionally diverse cytochrome P450s

Patrick J. Bakkes[1], Jan L. Riehm[2], Tanja Sagadin[3], Ansgar Rühlmann[1], Peter Schubert[1], Stefan Biemann[1], Marco Girhard[1], Michael C. Hutter[2], Rita Bernhardt[3] and Vlada B. Urlacher[1]*

[1] Institute of Biochemistry, Heinrich Heine University Düsseldorf, Universitätsstr. 1, 40225 Düsseldorf, Germany

[2] Center for Bioinformatics, Saarland University, Campus Building E2.1, 66123 Saarbrücken, Germany

[3] Institute of Biochemistry Saarland University, Campus Building B2.2, 66123 Saarbrücken, Germany

[*] Corresponding author: Vlada B. Urlacher (vlada.urlacher@uni-duesseldorf.de)

METHODS

Bacterial strains, commercial enzymes and chemicals - *E. coli* DH5α (Invitrogen, Karlsruhe, Germany) was used for molecular cloning and *E. coli* BL21(DE3) (Novagen, Darmstadt, Germany) was used for recombinant gene expression. Bacteria were grown in LB medium containing 100 µg/ml ampicillin. Molecular cloning and microbiological procedures were carried out by standard procedures[1]. Oligonucleotides were obtained from Eurofins MWG Operon (Ebersberg, Germany). Phusion DNA polymerase, Restriction enzymes, FastAP thermosensitive alkaline phosphatase, T4 DNA ligase and isopropyl-β-D-thiogalactopyranoside (IPTG) were obtained from Thermo Fisher Scientific (St. Leon-Rot, Germany). Glucose-6-phosphate dehydrogenase from *Saccharomyces cerevisiae* was purchased from Roche Diagnostics (Mannheim, Germany). NADPH was from Carbosynth (Berkshire, UK), whereas all other chemicals were purchased from Sigma-Aldrich (Schnelldorf, Germany).

Construction of the ykuN:fpr fusion gene - The primers used for the construction of *ykuN:fpr* are listed in Table S1. First *ykuN* and *fpr* were amplified separately using primer pairs 1-2 and 3-4, respectively (Table S1). Primer 1 introduced an N-terminal His_6-tag and simultaneously eliminated an internal *Nde*I restriction site at the 5' end of *ykuN* via a silent point mutation (T → **C**). The obtained PCR products were gel purified, mixed in a 1:1 ratio, and then subjected to a second round of PCR in the absence of additional primers. After 6 cycles, primers 1 and 4 were added to the mixture and PCR was allowed to continue for an additional 30 cycles. The *ykuN:fpr* fusion gene was then gel purified and subsequently cloned into pET11a (Novagen) using *Nde*I and *Bam*HI, yielding pET11a-*hYR*. DNA sequencing (GATC-Biotech, Konstanz, Germany) confirmed that the sequence of *ykuN:fpr* was correct.

Supplementary Table S1. PCR primers used to fuse *ykuN* to *fpr*. *Underlined nucleotides indicate restriction sites relevant to cloning; NdeI (CATATG) and BamHI (GGATCC), and linker insertion; NcoI (CCATGG). The overlapping primer pair 2-3 are fully complementary and lack the ykuN stop codon, which allows the in frame fusion of ykuN to fpr, with nucleotides of fpr highlighted in grey. To generate the NcoI site for DuaLinX insertion three extra nucleotides coding for Ala were incorporated at the fusion site of ykuN:fpr. As a result the DNA at the fusion site of the ykuN:fpr (YR) construct codes for -Ala-Met-Ala-, with minimal disturbance of the natural amino acid sequences.*

Primer (name)	Nucleotide sequence
1 (*ykuN-Fw*)	5'AGGAATTCCATATGCATCATCATCATCATCATGCTAAAGCCTTGATTACATA**C**GCCAG-3'
2 (*ykuN:fpr-Rv*)	5'-CTGTTACCCAATCAGCCATGGCTGAAACATGGATTTTTTCCT-3'
3 (*ykuN:fpr-Fw*)	5'-AGGAAAAAATCCATGTTTCAGCCATGGCTGATTGGGTAACAG-3'
4 (*fpr-Rv*)	5'-CAGCCGGATCCTTACCAGTAATGCTCC-3'

Linker insertion, ligation and transformation **-** Linker insertion between the fusion partners was carried out using the DuaLinX procedure as described in [2]. The DuaLinX are double stranded DNA elements of different lengths with *Nco*I-compatible overhangs, which depending on their orientation after insertion code for either flexible $(GGGGS)_n$ or rigid $([E/L]PPPP)_n$ linkers. Template pET11a-*hYR* was linearized with *Nco*I, dephosphorylated and mixed with desired DuaLinX at a 1:3 molar ratio. Ligation and transformation was carried out as described in [2]. The obtained constructs were verified by DNA sequencing (GATC, Konstanz, Germany). In such manner a set of 10 fusion constructs harbouring linkers of 5, 10, 15, 20 and 25 residues was created (Table S2). To study expression, the different fusion constructs were used for transformation of chemi-competent *E. coli* BL21(DE3) cells.

Table S2. Linkers used to connect YkuN and Fpr using the DuaLinX tool [2].

Linker	Length	Amino acid sequence
G1	5	GGGGS
P1	5	EPPPP
G2	10	GGGGS-GGGGS
P2	10	EPPPP-LPPPP
G3	15	GGGGS-GGGGS-GGGGS
P3	15	EPPPP-EPPPP-LPPPP
G4	20	GGGGS-GGGGS-GGGGS-GGGGS
P4	20	EPPPP-LPPPP-LPPPP-EPPPP
G5	25	GGGGS-GGGGS-GGGGS-GGGGS-GGGGS
P5	25	EPPPP-EPPPP-LPPPP-LPPPP-LPPPP

Expression and purification of YkuN-Fpr fusion proteins **-** Cryo-cultures of *E. coli* BL21(DE3) cells carrying the different fusion constructs were used to inoculate LB_{amp} medium at an OD_{600} of 0.12. Cultures were grown till an OD_{600} ~0.6 at 25°C and 140 rpm. Expression was then induced by the addition of IPTG (0.1 mM) and carried out for 20 h. The cells were subsequently harvested by centrifugation (8,000x g, 15 min, 4°C). After removal of the supernatant, the cell pellets were suspended in 8 ml buffer containing Tris-HCl, pH 7.5 (50 mM) and PMSF (0.1 mM). Cells were lysed by sonication on ice applying four cycles of 1 min sonication followed by 1 min intermission and the lysate was cleared by centrifugation (20,000x g, 30 min, 4°C). The supernatant was supplemented with FAD (10 µM) and FMN (10 µM) and incubated on ice for 1 h. To 4 volumes of this protein sample, 1 volume of 5x IMAC buffer containing Tris-HCl, pH 7.5 (250 mM) and NaCl (750 mM) was added and filtered through a 0.45 µm filter. The filtered protein samples were then applied to self-cast Ni^{2+}-NTA (Qiagen, Hilden, Germany) columns with

a 2 ml bed volume that were equilibrated with 1x IMAC buffer, which contains Tris-HCl, pH 7.5 (50 mM) and NaCl (150 mM). Non-specifically bound proteins were removed by using 1x IMAC buffer supplemented with 10 mM imidazole, while subsequent elution of the fusion proteins was carried out with 1x IMAC buffer supplemented with 150 mM imidazole. Immediately after elution the elution fractions (~8 ml) were 1:1 diluted with 1x IMAC buffer and then concentrated to a volume of ~1 ml using centrifugal devices (Sartorius, Vivaspin 15, 30 kDa MWCO). Residual imidazole was removed by using gravity flow PD midiTrap G-25 columns (GE-Healthcare) equilibrated in 1x IMAC buffer supplemented with 5% (v/v) glycerol. Finally, the purified fusion proteins were concentrated as described above and stored at -20°C.

Expression and purification of Fpr and FldA from *E. coli* were conducted as described in [2] and procedures for YkuN and CYP109B1 from *B. subtilis* were carried out as described in [3] and [4], respectively. *Thermobifida fusca* YX CYP154E1, *B. megaterium* CYP106A2 and bovine CYP21A2 were expressed and purified according to [5], [6] and [7] respectively, whereas bovine AdR and Adx were isolated as described in [8] and [9]. Crystal structures of CYP109B1 [10], CYP106A2 [11], CYP21A2 [12] have recently been reported.

Determination of protein concentration **-** The concentration of purified P450 monooxygenases was determined using the CO-difference spectral assay with $\varepsilon_{450-490}$ = 91 $mM^{-1}cm^{-1}$ [13]. The concentrations of Fpr, FldA and YkuN were calculated from their respective molar extinction coefficients ε_{456} = 7,100 $M^{-1}cm^{-1}$ [14], ε_{465} = 8,420 $M^{-1}cm^{-1}$ [15] and ε_{461} = 10,000 $M^{-1}cm^{-1}$ [16]. For the concentration determination of the fusion proteins a similar approach as described in [2] was followed. In brief, from absorbance spectra of known concentrations of YkuN, the ε_{456} was determined as 9,810 $M^{-1}cm^{-1}$. The extinction coefficient of the different YkuN-Fpr fusion constructs was then calculated by addition of the ε_{456} of Fpr and YkuN, yielding ε_{456} = 16,910 $M^{-1}cm^{-1}$ for the fusion proteins. Typical absorbance spectra are shown in Supplementary Figs. S2 and S3. Alternatively, the total protein concentration was determined by a Bradford-based assay [17] using bovine serum albumin as a standard.

GC-MS and HPLC analysis **-** CYP109B1 reaction mixtures were extracted two times with 0.8 ml diethyl ether and dried by the addition of anhydrous $MgSO_4$. The dried organic phase was evaporated and the residue was dissolved in 80 µl *N,O*-bis(trimethylsilyl)trifluoroacetamide containing 1% (v/v) trimethylchlorosilane for derivatization. Samples were transferred into GC vials and incubated at 80°C for 30 min. Myristic acid conversion was analyzed on a GC/MS-QP2010 (Shimadzu, Duisburg, Germany) equipped with a FS-Supreme-5 column (30 m x 0.25 mm x 0.25 µm, CS-Chromatographie Service GmbH, Langerwehe, Germany) as described previously [18]. CYP154E1 - After phase separation by centrifugation, the organic phase was analyzed for β-ionone conversion on the same GC-MS system as described above.

Samples were injected at a temperature of 250°C. The column temperature was set to 130°C, which was held for 2 min and then increased to 240°C at 20°C min^{-1}. Temperature was maintained for another 2 min and then further increased to 300°C at 50°C min^{-1} and held for 1 min. CYP106A2/CYP21A2 – Prior to HPLC analysis samples were dissolved in 200 µl of 10% (v/v) acetonitrile. HPLC experiments were performed on a Jasco system containing a PU-2080Plus Intelligent HPLC Pump, an AS-2050Plus Intelligent Sampler, an UV-2075Plus Intelligent UV/Vis Detector, a LG-2080-02 Ternary Gradient Unit, a DG-2080-53 3-Line Degasser, and a LC-NetII/ADC (Jasco, Gross-Umstadt, Germany). The HPLC system was equipped with a reversed phase ec MN Nucleodor C18 (5 µm, 4.0 x 125 mm) column (Macherey-Nagel, Düren, Germany) maintained at 40°C by a column thermostat (W.O. electronics, Austria). A mixture of acetonitrile and water (10:90) served as mobile phase, while flow rate was set to 1 ml/min. Compound separation was accomplished by mixing solvent A (water containing 10 % (v/v) acetonitrile) with solvent B (100 % acetonitrile). First a linear gradient from 11 % solvent B (and 89 % solvent A) to 33 % solvent B within 2 min was applied and then held at 33% for 4 min. Hereafter, the concentration of solvent B was increased to 100 % in 4 min and then kept at 100 % for 2 min. Finally, the column was re-equilibrated by employing a linear gradient to 11 % solvent B within 0.1 min, which was maintained for 3 min. For each measurement, 10 µL of sample was injected and absorbance was monitored at 240 nm.

Molecular dynamics (MD) simulations **-** To gain insight in the structural properties of the peptide linkers used for joining the redox partners in a physiological medium, molecular dynamics simulations were carried out in water as explicit solvent. Three-dimensional models for the glycine-rich (G1 - G5) and proline-rich (P1 - P5) linkers were generated using HYPERCHEM (version 6.02, Hypercube Inc., Gainsville, FL, 1999). The initial conformation of the G-linkers corresponded to a beta-sheet conformation, whereas the P-linkers were modelled as PP-helix II (φ = –75°, ψ = 150°). These initial conformations were chosen in order to start with the maximum possible length of the linkers, while simultaneously possessing an ordered structure that corresponds to an energetic minimum. Moreover, this approach allows monitoring changes in linker length during the simulation by inspecting the corresponding radius of gyration. The ends of the linkers were capped with acetyl (ACE) residues at the N-terminus and N-methylamine (NME) residues at the C-terminus. Each linker molecule was placed into a cubic box (with a scale to match the linker's length plus an additional 2 nm) of TIP3P (transferable intermolecular potential 3 point) waters [19] followed by a steepest descent energy minimization that was stopped at a gradient norm lower than 1000 kJ mol^{-1} nm^{-2}. Prior to the actual molecular dynamics simulations we equilibrated the linker sequences in two steps. The first step was carried out in a NVT (constant number of particles, volume and temperature) ensemble for 100 ps maintaining the temperature at 300 K using a modified Berendsen thermostat [20]. In the second step, the pressure of the system was

stabilized at 1 bar applying a Parinello-Rahman barostat [21] for 100 ps of NPT (constant number of particles, pressure and temperature) equilibration. All bonds in both steps were constrained by the LINCS [22] algorithm. Subsequently, regular MD production runs were carried out for 20 ns for each linker construct. Hereby Newton's equations of motion were integrated by the leapfrog algorithm at time steps of 2 fs. Subsequent analysis included investigation of plots of the radius of gyration (Rg) for the different linker types and lengths. Rg was calculated by the root mean square distance of atoms in the linker molecule from the center of mass.

RESULTS

1. Expression and purification of the redox fusion enzymes.

Expression and purification of the fusion proteins were carried out as described above. After IMAC and removal of imidazole, the fusion proteins were analysed by 12.5% SDS-PAGE (Fig. S1).

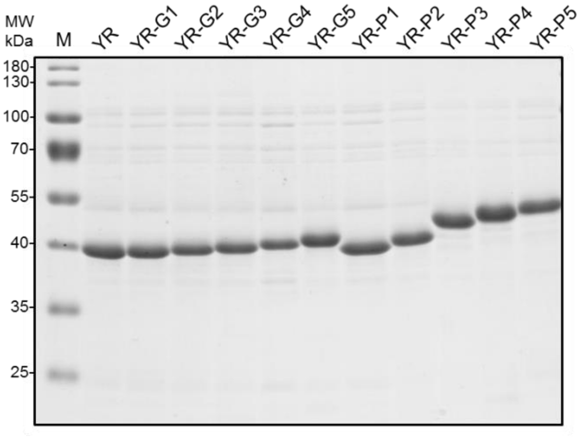

***Figure S1. SDS-PAGE analysis of IMAC purified YkuN-Fpr fusion proteins**. Fusion protein (2 µg) was loaded in each lane. Indicated are YkuN-Fpr fusion construct without linker (YR) and constructs carrying flexible glycine-rich linkers G1 - G5 or rigid proline-rich linkers P1 – P5 (for linker specifications see Table S2 [2]).*

2. Spectroscopic analysis of the fusion proteins: increased stability of Fpr upon fusion to YkuN.

Protein stability of the P450s and dedicated redox partner(s) can be limiting in P450-catalyzed reactions. Previous experiments have shown that protein stability of YkuN/Fpr in CYP154A8-catalyzed production of chiral 2-alcohols is a limiting factor especially in prolonged (~18 h) conversion reactions [23]. Moreover, pioneering work involving the biochemical characterization of *E. coli* Fpr revealed that this protein slowly precipitates during spectral analysis [14]. Consistent with this observation, we noted that purified Fpr forms visible aggregates upon dilution into buffer (50 mM Tris-HCl pH 7.5), as illustrated by the overall increase in absorbance over time due to light scatter (Fig. S2B). In contrast, under the same conditions, both YkuN and the YkuN-Fpr fusion constructs exhibited no visible aggregation and the absorbance spectra remained virtually unchanged over time (Fig. S2A and C and data not shown). Thus in the absence of stabilizing agents (such as NaCl, glycerol or interacting protein partners) Fpr is prone to aggregation, whereas Fpr is apparently stabilized upon fusion to YkuN. Moreover, even after prolonged incubation (24 h) at room temperature the characteristic absorbance maximum at 460 nm (Fig. S3) of the fusion constructs remained virtually unchanged, with no evidence of protein precipitation.

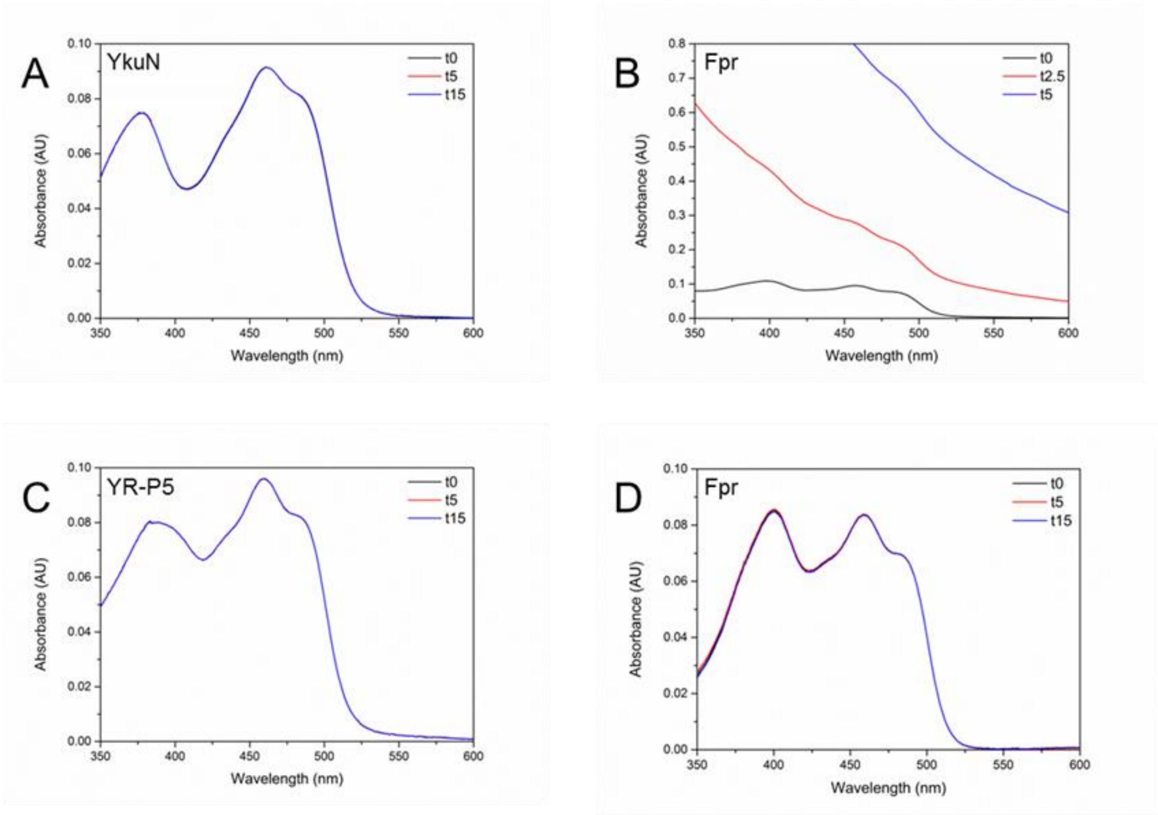

Figure S2. Stabilisation of Fpr upon fusion to YkuN. *Arbitrary amounts of indicated purified redox protein were diluted in appropriate buffer (**A - C**: 50 mM Tris-HCl pH 7.5, and **D**: 50 mM KPi pH 7.5, 500 mM NaCl, 20% v/v glycerol), typically yielding final concentrations in the range of 4 - 8 µM. At indicated time points (minutes after dilution), spectra were recorded at 25°C using a thermostated Perkin Elmer lambda 35 spectrometer. Spectra were corrected for buffer absorbance.*

3. Spectral properties of the YkuN-Fpr fusion constructs.

Consistent with previous observations for fully oxidized (quinone) FMN-containing YkuN [24], the absorbance spectrum of purified YkuN shows two major absorbance bands with maxima at 378 nm and 462 nm, with a shoulder around 480 nm (Fig. S3A). The spectral properties of purified (oxidized) FAD-containing Fpr are nearly identical to those reported previously [15,25], exhibiting characteristic peaks at 400 nm and ~458 nm, with a shoulder around 480 nm (Fig. S3A). The peak at 400 nm is a rather uncommon feature for flavoproteins, which typically show a maximum at ~380 nm. This unusual absorbance property of Fpr is thought to arise from a bent conformation of the FAD cofactor governed, in part, by the extra C-terminal Trp248 [26]. The linker-less YkuN-Fpr (YR) fusion construct exhibits the hallmarks of the individual redox partners combined. The absorbance spectrum of YR shows a rather broad peak between 378 – 400 nm, with a maximum around 389 nm, and a second maximum at 460 nm, with a shoulder around 480 nm (Fig. S3A). The depicted YkuN-Fpr fusion constructs carrying different linkers showed nearly identical absorbance spectra (Fig. S3B), which indicates that the fusion constructs have a similar cofactor content (FAD + FMN) and a similar protein environment of the cofactors.

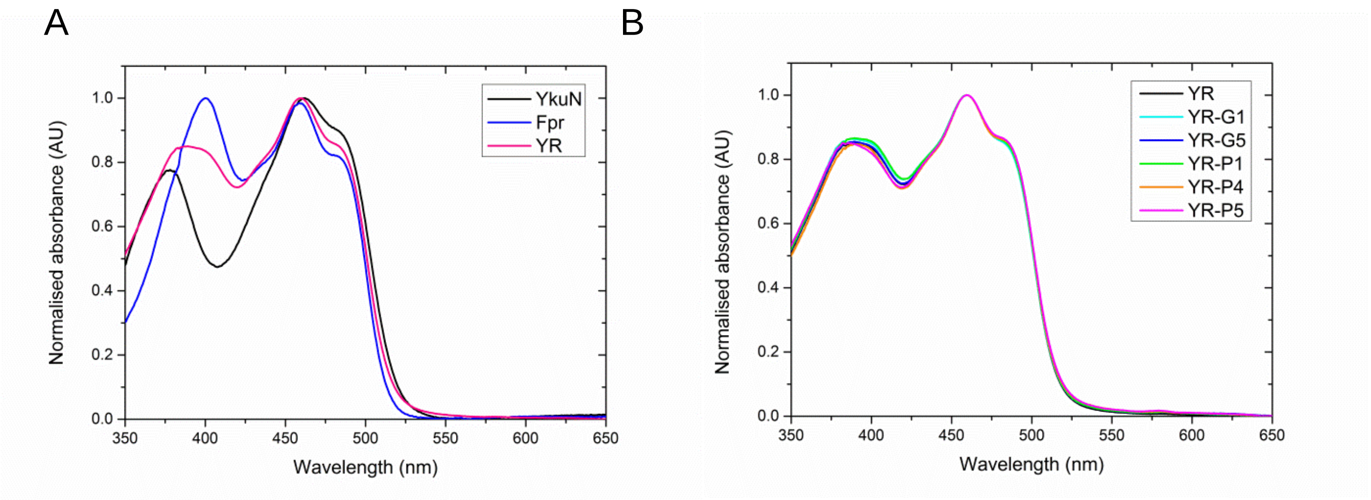

Figure S3. Spectral properties of the individual and fused redox partners, YkuN and Fpr. *Arbitrary amounts of indicated redox protein were diluted in appropriate buffer (**A**, 50mM KPi pH 7.5, 500 mM NaCl, 20% (v/v) glycerol, and **B**, 50 mM Tris-HCl pH 7.5), typically yielding final concentrations in the range of 4 – 8 µM. Spectra were recorded at 25°C using a thermostated Perkin Elmer Lambda 35 spectrometer. Presented normalised spectra are the average of 3 recorded spectra that were corrected for buffer absorbance.*

4. Reduction of the CYP109B1 heme-iron by various redox partners.

Reduction of the P450 heme-iron (Fe^{3+} to Fe^{2+}) was monitored by using a CO-difference spectroscopy-based method [13]. Heme-iron reduction was measured in the presence of carbon monoxide under anaerobic conditions at 20°C, using 1 µM CYP109B1 and 4 µM of indicated redox partner(s). Reactions were carried out as described in the Methods section of the main paper. Kinetic traces were obtained from the acquired absorbance spectra (340 - 700 nm), by plotting the $\Delta A_{450} - A_{490}$ against the time [27]. Kinetic data were fitted using equation $A = A_1(1 - e^{k_1 t}) + A_2(1 - e^{k_2 t}) + C$. Typical kinetic traces and corresponding fits (thin lines) are shown in Fig. S4.

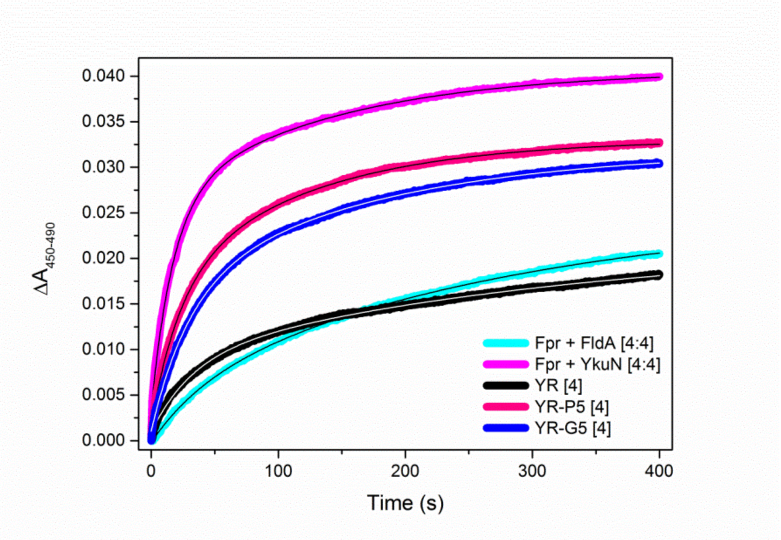

Figure S4. CYP109B1 heme-iron reduction by different redox partners.

5. Conversion of myristic acid by *B. subtilis* CYP109B1. Activity of the fatty acid hydroxylase CYP109B1[10] was determined in 200 µl reaction mixtures containing 50 mM Tris-HCl pH 7.5, 1 mM $MgCl_2$, 4 mM glucose-6-phosphate (G6P), 1 U *S. cerevisiae* G6P-dehydrogenase, 1 µM CYP109B1 and redox protein(s) as indicated (Supplementary Table S3)

Supplementary Table S3. Product distribution for the CYP109B1-catalysed conversion of myristic acid in the presence of different redox partners. *Myristic acid conversion data represent average values of at least three independent conversion reactions with indicated standard deviation. [1] Values in brackets indicate final concentrations in µM of the corresponding redox proteins. [2] In CYP109B1 reactions exhibiting high turnover (≥ 60% myristic acid conversion) an unidentified product appeared (retention time 9.50 min), which likely represents an overoxidation product of myristic acid.*

Redox protein(s) [1]	Myristic acid conversion (%)	Myristic acid product distribution (%)					
		ω_{-1}<br>10.37 min	**ω_{-2}<br>10.22 min**	**ω_{-3}<br>9.97 min**	ω_{-4}<br>9.80 min	ω_{-5}<br>10.72 min	X [2]<br>9.50 min
Fpr + FldA [1:1]	22.1 ± 4.2	16.5 ± 1.2	**29.7 ± 1.0**	**27.8 ± 0.8**	18.4 ± 1.0	7.6 ± 1.5	N.D.
Fpr + YkuN [1:1]	59.9 ± 12.5	19.0 ± 0.6	**31.7 ± 0.6**	**28.1 ± 0.7**	15.8 ± 0.3	4.6 ± 0.6	0.9 ± 0.5
Fpr + YkuN [1:10]	96.0 ± 0.5	14.0 ± 1.0	**26.2 ± 0.5**	**25.5 ± 0.2**	19.6 ± 0.5	5.4 ± 0.9	9.2 ± 1.6
Fpr + YkuN [4:4]	93.5 ± 1.8	17.6 ± 0.4	**29.3 ± 0.5**	**27.1 ± 0.4**	17.8 ± 0.2	4.2 ± 0.9	3.9 ± 0.9
YR [4]	37.5 ± 2.7	18.5 ± 0.5	**31.2 ± 1.2**	**27.8 ± 0.7**	15.4 ± 0.3	6.1 ± 2.3	N.D.
YR-G5 [4]	63.0 ± 4.9	19.4 ± 0.2	**32.1 ± 0.3**	**28.1 ± 0.4**	15.6 ± 0.3	3.8 ± 1.5	1.0 ± 0.1
YR-P5 [4]	86.1 ± 2.2	19.2 ± 0.2	**30.7 ± 0.2**	**27.4 ± 0.3**	16.1 ± 0.4	4.6 ± 0.8	2.0 ± 0.3

Supplementary Table S4. Coupling efficiency for CYP154E1-mediated conversion of β-ionone using different redox partners.

Reconstituted system	Respective stoichiometry (µM)	Coupling efficiency (%)
Fpr/YkuN/CYP154E1	1 : 10 : 1	16.7
	1 : 4 : 1	42.0
	4 : 4 : 1	44.2
YR-P5/CYP154E1	4 : 1	50.2
	1 : 1	44.8

Supplementary Table S5. Total turnover numbers for β-ionone achieved with CYP154E1 reconstituted systems.

Reconstituted system	Respective stoichiometry (µM)	Conversion (h)	TTN
Fpr/YkuN/CYP154E1	4 : 4 : 1	1	1439
	2 : 2 : 0.5	6	1948
YR-P5/CYP154E1	4 : 4 : 1	1	976
	2 : 2 : 0.5	6	1582

6. Conversion of myristic acid by *B. subtilis* CYP109B1 in the absence of NADPH regeneration

Activity of the fatty acid hydroxylase CYP109B1 was determined as described in Methods section of the main manuscript. Reaction mixtures (200 µl) in 96-microwell plate contained 50 mM Tris-HCl pH 7.5, 1 mM $MgCl_2$, 4 mM glucose-6-phosphate (G6P), 1 µM CYP109B1, 4 µM redox protein(s) as indicated (Fig. S5), 200 µM myristic acid and 200 µM NADPH.

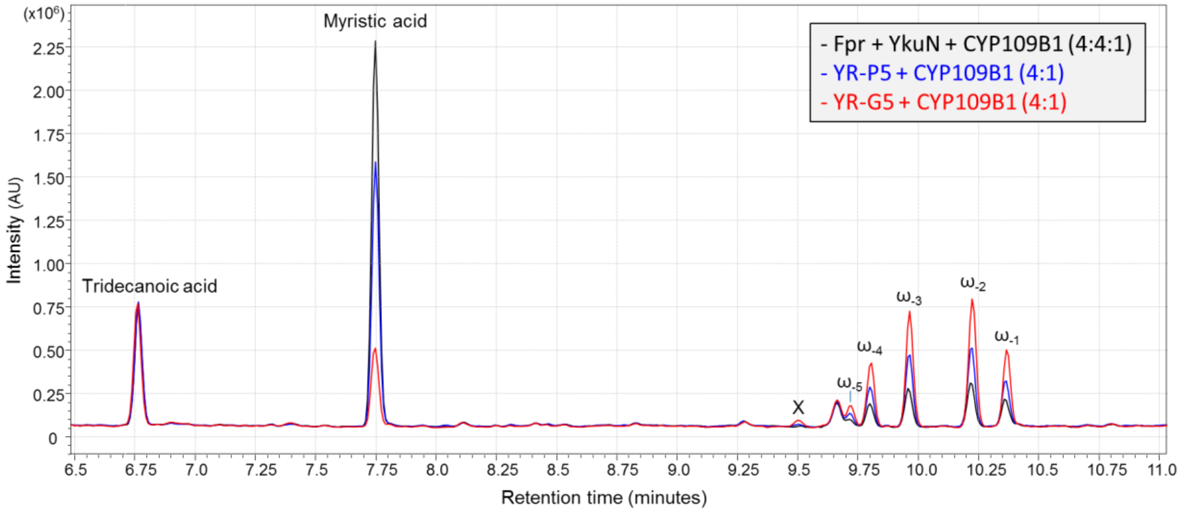

Figure S5. GC-MS analysis of CYP109B1 conversion reactions using different redox partners in the absence of G6P-dehydrogenase. *The employed ratio of NADPH:myristic acid was 1:1 (200 µM each, respectively). After NADPH depletion, the proportion of myristic acid that was converted was determined. Herein, the coupling efficiency equals the proportion (%) of myristic acid converted upon NADPH depletion. Tridecanoic acid (50 µM) was used as internal standard. Traces are shown for reactions performed with redox partners Fpr/YkuN (black), YR-P5 (blue) and YR-G5 (red), achieving 25%, 47% and 84% conversion, respectively. X indicates a possible overoxidation product of myristic acid, whereas the peak eluting at 9.67 min represents the fatty acid impurity hexadecanoic acid.*

REFERENCES

1 Sambrook, J. & Russell, D. W. *Molecular cloning: a laboratory manual, 3nd ed.*, (Cold Spring Harbor Laboratory Press, 2001).

2 Bakkes, P. J. *et al.* Design and improvement of artificial redox modules by molecular fusion of flavodoxin and flavodoxin reductase from *Escherichia coli*. *Sci. Rep.* **5**, 12158, doi:10.1038/srep12158 (2015).

3 Girhard, M., Klaus, T., Khatri, Y., Bernhardt, R. & Urlacher, V. B. Characterization of the versatile monooxygenase CYP109B1 from *Bacillus subtilis*. *Appl. Microbiol. Biotechnol.* **87**, 595-607, doi:10.1007/s00253-010-2472-z (2010).

4 Girhard, M. *et al.* Regioselective biooxidation of (+)-valencene by recombinant *E. coli* expressing CYP109B1 from *Bacillus subtilis* in a two-liquid-phase system. *Microb. Cell Fact.* **8**, 36 (2009).

5 von Bühler, C., Le-Huu, P. & Urlacher, V. B. Cluster screening: an effective approach for probing the substrate space of uncharacterized cytochrome P450s. *ChemBioChem* **14**, 2189-2198, doi:10.1002/cbic.201300271 (2013).

6 Lisurek, M., Kang, M. J., Hartmann, R. W. & Bernhardt, R. Identification of monohydroxy progesterones produced by CYP106A2 using comparative HPLC and electrospray ionisation collision-induced dissociation mass spectrometry. *Biochem. Biophys. Res. Commun.* **319**, 677-682 (2004).

7 Brixius-Anderko, S., Schiffer, L., Hannemann, F., Janocha, B. & Bernhardt, R. A CYP21A2 based whole-cell system in *Escherichia coli* for the biotechnological production of premedrol. *Microb. Cell Fact.* **14**, 135, doi:10.1186/s12934-015-0333-2 (2015).

8 Sagara, Y. *et al.* Direct expression of adrenodoxin reductase in *Escherichia coli* and the functional characterization. *Biol. Pharm. Bull.* **16**, 627-630 (1993).

9 Uhlmann, H., Beckert, V., Schwarz, D. & Bernhardt, R. Expression of bovine adrenodoxin in *E. coli* and site-directed mutagenesis of /2 Fe-2S/ cluster ligands. *Biochem. Biophys. Res. Commun.* **188**, 1131-1138 (1992).

10 Zhang, A. *et al.* The crystal structure of the versatile cytochrome P450 enzyme CYP109B1 from *Bacillus subtilis*. *Mol. Biosyst.* **11**, 869-881, doi:10.1039/c4mb00665h (2015).

11 Janocha, S., Carius, Y., Hutter, M., Lancaster, C. R. & Bernhardt, R. Crystal Structure of CYP106A2 in Substrate-Free and Substrate-Bound Form. *ChemBioChem* **17**, 852-860, doi:10.1002/cbic.201500524 (2016).

12 Haider, S. *et al.* Structure-phenotype correlations of human CYP21A2 mutations in congenital adrenal hyperplasia. *Proc. Natl. Acad. Sci. U. S. A.* **110**, 2605-2610, doi:10.1073/pnas.1221133110 (2013).

13 Omura, T. & Sato, R. The Carbon Monoxide-Binding Pigment of Liver Microsomes. I. Evidence for Its Hemoprotein Nature. *J. Biol. Chem.* **239**, 2370-2378 (1964).

14 McIver, L. *et al.* Characterisation of flavodoxin NADP+ oxidoreductase and flavodoxin; key components of electron transfer in *Escherichia coli*. *Eur. J. Biochem.* **257**, 577-585 (1998).

15 Fujii, K. & Huennekens, F. M. Activation of methionine synthetase by a reduced triphosphopyridine nucleotide-dependent flavoprotein system. *J. Biol. Chem.* **249**, 6745-6753 (1974).

16 Wang, Z. Q. *et al.* Bacterial flavodoxins support nitric oxide production by *Bacillus subtilis* nitric-oxide synthase. *J. Biol. Chem.* **282**, 2196-2202 (2007).

17 Bradford, M. M. A rapid and sensitive method for the quantitation of microgram quantities of protein utilizing the principle of protein-dye binding. *Anal. Biochem.* **72**, 248-254 (1976).

18 Girhard, M., Tieves, F., Weber, E., Smit, M. S. & Urlacher, V. B. Cytochrome P450 reductase from *Candida apicola*: versatile redox partner for bacterial P450s. *Appl. Microbiol. Biotechnol.* **97**, 1625-1635, doi:10.1007/s00253-012-4026-z (2013).

19 Jorgensen, W. L. & Madura, J. D. Quantum and Statistical Studies of Liquids .25. Solvation and Conformation of Methanol in Water. *J. Am. Chem. Soc.* **105**, 1407-1413, doi:DOI 10.1021/ja00344a001 (1983).

20 Berendsen, H. J. C., Postma, J. P. M., Vangunsteren, W. F., Dinola, A. & Haak, J. R. Molecular-Dynamics with Coupling to an External Bath. *J. Chem. Phys.* **81**, 3684-3690, doi:Doi 10.1063/1.448118 (1984).

21 Parrinello, M. & Rahman, A. Polymorphic Transitions in Single-Crystals - a New Molecular-Dynamics Method. *J. Appl. Phys.* **52**, 7182-7190, doi:Doi 10.1063/1.328693 (1981).

22 Hess, B. P-LINCS: A Parallel Linear Constraint Solver for Molecular Simulation. *J. Chem. Theory Comput.* **4**, 116-122, doi:10.1021/ct700200b (2008).

23 von Bühler, C. J. & Urlacher, V. B. A novel P450-based biocatalyst for the selective production of chiral 2-alkanols. *Chem. Commun. (Camb.)* **50**, 4089-4091, doi:10.1039/c4cc00647j (2014).

24 Lawson, R. J., von Wachenfeldt, C., Haq, I., Perkins, J. & Munro, A. W. Expression and characterization of the two flavodoxin proteins of *Bacillus subtilis*, YkuN and YkuP: biophysical properties and interactions with cytochrome P450 BioI. *Biochemistry* **43**, 12390-12409 (2004).

25 Jenkins, C. M. & Waterman, M. R. NADPH-flavodoxin reductase and flavodoxin from *Escherichia coli*: characteristics as a soluble microsomal P450 reductase. *Biochemistry* **37**, 6106-6113 (1998).

26 Ingelman, M., Bianchi, V. & Eklund, H. The three-dimensional structure of flavodoxin reductase from *Escherichia coli* at 1.7 A resolution. *J. Mol. Biol.* **268**, 147-157, doi:10.1006/jmbi.1997.0957 (1997).

27 Honda Malca, S., Girhard, M., Schuster, S., Durre, P. & Urlacher, V. B. Expression, purification and characterization of two *Clostridium acetobutylicum* flavodoxins: Potential electron transfer partners for CYP152A2. *Biochim. Biophys. Acta* **1814**, 257-264, doi:10.1016/j.bbapap.2010.06.013 (2011).
